# Dscam1 Forms a Complex with Robo1 and the N-Terminal Fragment of Slit to Promote the Growth of Longitudinal Axons

**DOI:** 10.1371/journal.pbio.1002560

**Published:** 2016-09-21

**Authors:** Maryam Alavi, Minmin Song, Gracie L. Andrews King, Taylor Gillis, Robert Propst, Matthew Lamanuzzi, Adam Bousum, Amanda Miller, Ryan Allen, Thomas Kidd

**Affiliations:** Department of Biology, University of Nevada, Reno, Nevada, United States of America; Columbia University Medical Center, UNITED STATES

## Abstract

The Slit protein is a major midline repellent for central nervous system (CNS) axons. In vivo, Slit is proteolytically cleaved into N- and C-terminal fragments, but the biological significance of this is unknown. Analysis in the *Drosophila* ventral nerve cord of a *slit* allele (*slit-UC*) that cannot be cleaved revealed that midline repulsion is still present but longitudinal axon guidance is disrupted, particularly across segment boundaries. Double mutants for the Slit receptors *Dscam1* and *robo1* strongly resemble the *slit-UC* phenotype, suggesting they cooperate in longitudinal axon guidance, and through biochemical approaches, we found that Dscam1 and Robo1 form a complex dependent on Slit-N. In contrast, Robo1 binding alone shows a preference for full-length Slit, whereas Dscam1 only binds Slit-N. Using a variety of transgenes, we demonstrated that Dscam1 appears to modify the output of Robo/Slit complexes so that signaling is no longer repulsive. Our data suggest that the complex is promoting longitudinal axon growth across the segment boundary. The ability of Dscam1 to modify the output of other receptors in a ligand-dependent fashion may be a general principle for Dscam proteins.

## Abstract 2

During the development of the insect central nervous system, cleavage of the secreted signaling protein Slit allows the formation of a complex with the receptors Dscam1 and Robo1 that is required for longitudinal axon growth across body segment boundaries.

## Author Summary

Most complex nervous systems have an anterior-posterior axis of symmetry: the midline of the central nervous system. Longitudinal nerves, formed by bundled axons, connect the brain to other parts of the body and grow long distances parallel to the central nervous system midline. In the fruit fly, the nerve cord is segmented, and the boundary of each segment represents a challenge for the longitudinal axons to cross. What cues promote growth of axons across these boundaries remains unknown. The Slit protein is classically known as a repulsive signal that prevents axons from crossing the midline by binding to its receptor Robo on the surface of axons. However, it is known that Slit is cleaved into two fragments, but the significance of this process remains to be elucidated. In this study, we find that cleavage of Slit allows the large fragment (Slit-N) to bind to another receptor called Dscam1, which then forms a complex with Robo1. Our experiments confirm that Slit processing is required for the longitudinal growth of axons across the segment boundary. Our work suggests that the Slit-N-Dscam1-Robo1 complex signals axons to grow, but Slit-N is not a specific navigational cue. Dscam proteins have evolutionarily conserved ligand-binding activities; therefore, they may be able to alter the signaling output of other receptors. The Slit fragments play diverse roles, from tumor metastasis to HIV replication; thus, identifying a receptor that is specific for Slit-N provides a molecular explanation for why cleavage occurs.

## Introduction

Longitudinal axon guidance is distinguished by long periods of growth independent of intermediate targets. In vertebrates, long distance gradients of Wnt and Shh have been shown to guide longitudinal axons in an anterior-posterior direction [1,2]. Longitudinal axons also respond to local cues derived from the central nervous system (CNS) midline, notably attractants such as Netrin and repellents such as Slit. The conflicting actions of these cues act to set the dorsal-ventral positions for longitudinal axon pioneers and dopaminergic axons [3–6]. In vitro culture of longitudinal explants with both Netrin and Slit synergistically promotes axon growth [5], suggesting that the opposing cues not only define accurate lateral positioning but also may promote axon growth.

**Fig 1:**
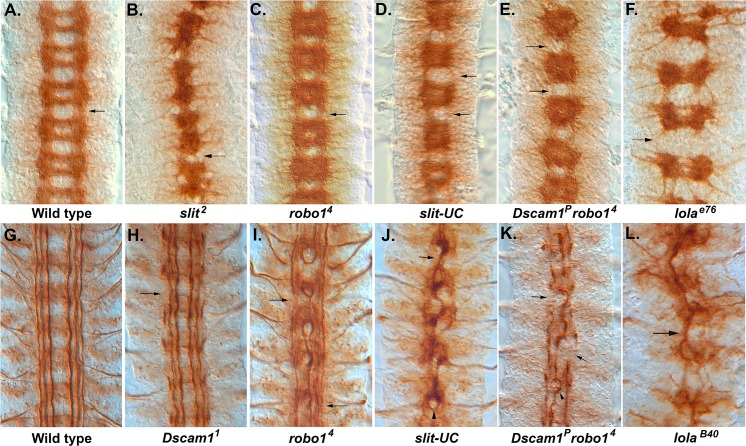
Longitudinal axon guidance in *Dscam1*, *robo1*, and *slit-UC* mutants. Embryos stained with monoclonal antibody (mAb) BP102 (A–D) to reveal the central nervous system (CNS) axon scaffold and anti-Fas2 (1D4) to reveal the longitudinal tracts (E–L). (A) Stage 16 wild-type embryo with an intersegmental longitudinal tract marked by an arrow. (B) *slit*^*2*^ homozygous embryo displaying the characteristic collapse of the axon scaffold onto the CNS midline. (C) *robo1*^*4*^ homozygous embryo showing the characteristic pattern of thickened commissures and reduced longitudinals (arrow). BP102 staining in the longitudinal tracts is reduced. (D) Stage 16 uncleavable *slit* mutant displaying reduced or absent longitudinals (arrows) and variability in the degree of axon collapse towards the midline in each segment, e.g., a gap between the commissures is visible in the lowest segment but not in any other segment. (E) *Dscam1*^*P*^ *robo*^*4*^ mutant in which several longitudinal tracts are absent (arrows). The nerve cord has not condensed as much as wild type or the *robo* mutant, so less segments are visible in the field of view. The individual segments are more variable than the *robo* mutant. (F) *lola*^*e76*^, a strong allele in which the longitudinal connectives (arrow) are almost completely absent. There is also a reduction in the thickness of the commissures. (G) Stage 17 wild-type embryo with longitudinal tracts running parallel to the CNS midline (center of image). (H) *Dscam1*^*1*^ embryo with breaks to the outermost fascicle (arrow) and an overall waviness to the longitudinal fascicles. (I) *robo1*^*4*^ mutant in which the innermost fascicles frequently merge and form circles around the midline. The medial and lateral fascicles are present (not always in the focal plane) and largely intact but display thinning and occasional breaks and defasciculation (arrows). (J) *slit-UC* mutant in which many of the longitudinal axons have fused to form a large fascicle that meanders along the midline (arrow). Remnants of the outermost fascicle are present but slightly out of focus, reflecting an overall degree of disorganization to the nerve cord. The occasional *robo-*like circle is visible (arrowhead). (K) *Dscam1*^*P*^ *robo*^*4*^ homozygous embryo displaying disruption to all the longitudinal axon tracts. A large, thick fascicle follows the midline, occasionally forming circles (arrowhead). The outermost fascicle is still present but is thicker than normal, so it has likely fused with the intermediate fascicle. There are frequent breaks, and sometimes the fascicle is completely absent (arrows). (L) *lola*^*B40*^ strong allele showing a large fascicle wandering back and forth across the midline (arrow). The underlying data are shown in S1 Data.

In *Drosophila*, the highly organized and segmented nature of the nerve cord makes it easy to detect gross defects in longitudinal axon guidance [7]. Mutations in the *lola* gene specifically disrupt longitudinal formation between segments (Fig 1F). Lola is a transcription factor that regulates the expression of multiple axon guidance genes such as *robo1*, *slit*, and *Dscam1* [8,9]. *Notch* and *Delta* are the only two cell surface genes identified that have highly penetrant longitudinal disruption phenotypes as single mutants [10,11]. Notch signaling induces neurons to produce a mesh of projections that link segments, providing a substrate for navigating growth cones [12]. The Netrin receptor Frazzled (Fra) is found on this mesh, as well as axons, and traps Netrin diffusing from the CNS midline [13]. Longitudinal pioneer axons grow along the edge of this Netrin-positive region [14]. Netrin therefore has two activities in longitudinal axon guidance as a local contact-dependent cue and as a midline-derived chemoattractant that must be suppressed by other signals to prevent inappropriate midline crossing.

The Robo/Slit signaling system plays a key role in defining the lateral position of longitudinal axons in *Drosophila*, acting as a repellent from the CNS midline [15–18]. The emerging view is that Slit and Netrin signaling balance each other to maintain the lateral position of axons, and this view is supported by mutant analysis in flies and vertebrates [5,6,14,19]. However, in embryos with reduced Robo/Slit signaling in which longitudinal axons inappropriately cross the midline, ectopic *slit* expression in the longitudinal pathway was sufficient to rescue guidance [14]. Nondirectional *slit* signals can therefore promote longitudinal trajectories by preventing midline crossing, suggesting that Robo/Slit signaling suppresses the Netrin-induced attraction to the midline [14]. The results of subsequent epistasis experiments examining the behavior of the pCC longitudinal pioneer axon in combinations of *fra*, *robo1*, and *slit* mutants are consistent with this conclusion [19]. These observations lead to the question of whether this secondary Slit activity is mediated by Robo or alternative receptors.

Dscam1 is a high-affinity receptor for Slit with a role in axon branching of adult mechanosensory axons [20]. In the embryo, *Dscam1* mutants have strong disruptions to the outermost longitudinal fascicle, and intact fascicles often have a wavy appearance suggesting a role in longitudinal growth and guidance [21]. We find that Dscam1 only binds Slit-N and that binding recruits Robo1 in a complex. A *slit* mutant that cannot be cleaved (*slit-UC*) [22] retains repulsive activity but displays strong disruption of longitudinal axon guidance, suggesting that Slit-N is required for the progression of longitudinal axons from segment to segment. Several lines of genetic evidence suggest that Slit-N signaling through the Dscam1-Robo1 complex promotes longitudinal axon outgrowth across the segment boundary.

## Results

### Analysis of an Uncleavable *slit* Mutation Reveals a Role in Longitudinal Axon Guidance

Proteolytic cleavage of Slit does not appear to be required for axon repulsion, as expression of an uncleavable *slit* transgene rescues *slit* mutants as effectively as wild-type *slit* [23]. Subsequent analysis of a *slit* allele (*slit-UC*) incapable of being cleaved revealed a role for Slit cleavage in muscle migration [22]. We analyzed the effect of the *slit-UC* allele on CNS formation and found that the axon scaffold superficially resembles that of a *robo1* mutant (Fig 1C and 1D). However, while *robo1* mutants have a highly regular CNS axon scaffold, *slit-UC* mutants are variable from segment to segment. The *slit-UC* longitudinal connectives between segments are often missing or have collapsed into one tract, whereas *robo1* mutants almost always have two tracts on either side of the midline [7]. To analyze this phenotype in more detail, we stained with anti-Fasciclin 2 (Fas2), which labels three parallel longitudinal tracts on either side of the midline. In the *slit-UC* mutant, all three longitudinal fascicles contribute to form a single thick fascicle that meanders along the midline forming occasional circles of axons (Fig 1J). The outermost fascicle is highly discontinuous and frequently appears to have stalled. This contrasts with the *robo1* phenotype in which only the innermost (pCC/MP1) pathway wanders across the midline, while the medial and lateral tracts generally do not, although they can be thinner or show occasional breaks (Fig 1I). The *slit-UC* allele therefore has a phenotype that is stronger than *robo1* mutants alone. The *slit-UC* Fas2 phenotype resembles strong *lola* alleles in which the majority of Fas2-positive axons wander back and forth across the midline in a single large fascicle (Fig 1L) [8].

### *Dscam robo1* Double Mutants Resemble the *slit-UC* Phenotype

**Fig 2:**
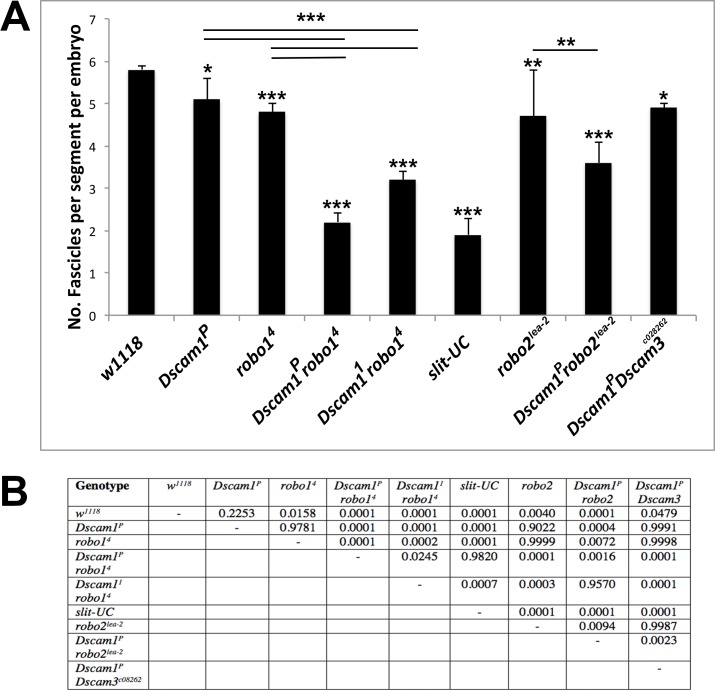
Statistical analysis of longitudinal axon guidance phenotypes. For each of the mutant genotypes shown, the number of fascicles visible at the segment boundaries was counted in stage 17 embryos stained with anti-Fas2. The number of fascicles was divided by the number of segments scored in the embryo to get an average number of fascicles per segment, which is six in wild type (S1 Data). *w*^*1118*^ was used as wild type. (A) Graph displaying the results of quantification. The data were analyzed by using a Tukey honest significant difference (HSD) within a one-way ANOVA. Statistical significant differences relative to wild type are shown above each individual column (* *p* < 0.05, ** *p* < 0.01, *** *p* < 0.0001). Select pairwise comparisons are shown as horizontal bars. Error bars are one standard deviation. (B) Complete results of the ANOVA showing the significance of all pairwise comparisons. The underlying data are shown in S1 Data.

As the *slit-UC* phenotype is more severe than *robo1*, we constructed double mutants for both *Dscam1* and *robo1* to see if any synergy between these Slit receptors was observed. *Dscam1 robo1* double mutants have an enhanced *robo1* phenotype resembling that of *slit-UC* because the longitudinal connectives are frequently reduced to one tract (Fig 1E). Unlike *slit-UC*, which appears variable at all stages of axon outgrowth, *Dscam1 robo1* double mutants are relatively regular until stage 16 and then rapidly become more irregular perhaps because the nerve cord often fails to condense (S1 Fig). We stained the longitudinal tracts with anti-Fas2 and found that in *Dscam1 robo1* mutants, all the axon tracts are disrupted, with a single thick fascicle wandering across the midline, and the medial and lateral tracts are clearly disrupted, frequently stalled so there is no connection of segments (Fig 1K). This phenotype strongly resembles the *slit-UC* phenotype. To accurately quantify longitudinal phenotypes, we counted the number of Fas2-positive fascicles at the segment boundaries (S1 Data). We used two different *Dscam1* alleles to generate the double mutants, and one combination (*Dscam1*^*P*^ *robo*^*4*^) was statistically indistinguishable from *slit-UC* (*p* = 0.98, Tukey honest significant difference [HSD] test, Fig 2). The other combination (*Dscam1*^*1*^ *robo*^*4*^) had a weaker phenotype, although it was still significantly stronger than any of the other genotypes tested. This may be due to the nature of the alleles or genetic modifiers on the mutant chromosome. Both *Dscam1* and *robo1* mutants have breaks in the outermost Fas2-positive fascicle (Fig 1H and 1I), and the double mutant combination had a statistically significant increase in severity of the longitudinal axon guidance phenotype (*p* < 0.0001, Tukey HSD test, Fig 2). Mutants in the *robo2* and *Dscam3* paralogues have disruptions in the longitudinal fascicles (S2 Fig), so we included these genes in the fascicle analysis, finding they make minor contributions (Fig 2). In summary, Slit cleavage and the *Dscam1* and *robo1* genes are all required for longitudinal axon guidance, with the high frequency of stalled (missing) Fas2-positive tracts suggesting an axon growth defect as well.

### Pioneer Axon Basis of the *slit-UC* Phenotype

**Fig 3:**
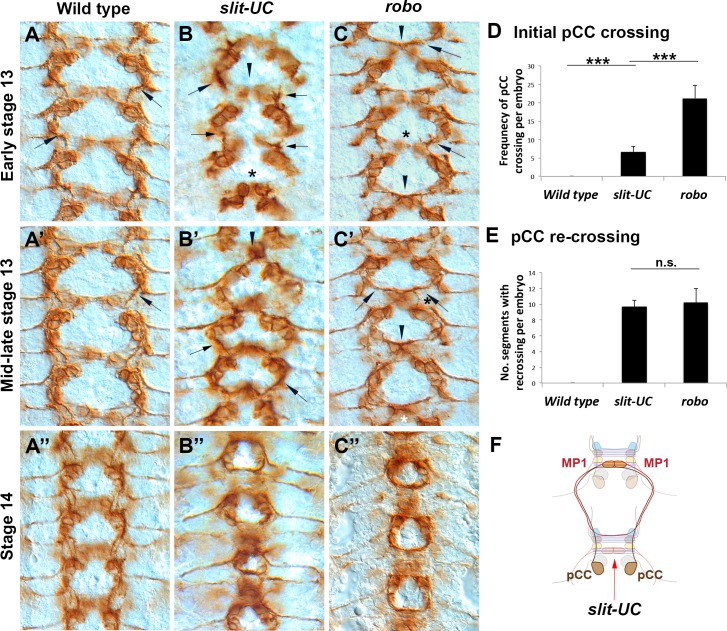
Analysis of longitudinal pioneer axons in an uncleavable *slit* mutant. Anti-Fasciclin 2 (mAb 1D4) staining of embryos during early CNS development (stages 13–14 as indicated). The anterior is to the top of the image. (A) Wild-type embryo in which the pCC growth cones (arrows) are growing anteriorly and away from the midline. (B) A *slit-UC* mutant in which the pCC growth cones (arrows) are growing anteriorly and away from the midline (arrowhead), resembling wild-type behavior. However, the growth cones are growing at different rates. The cell bodies are also positioned closer to the midline, particularly in the lowest segment (asterisk). (C) *robo*^*4*^ mutant in which the pCC growth cones (arrows) have grown towards the midline and fasciculated with their contralateral homologues (arrowheads). The asterisk marks a relatively rare occurrence in which the pCC axons failed to cross the midline. (A′) Mid-stage 13 wild-type embryo in which the pCC axons have met and fasciculated with descending longitudinal axon pioneers. (B′) *slit-UC* embryo in which most pCC axons have continuing growing ipsilaterally towards descending longitudinal axon pioneers (arrows), but in one segment, the pCC axons have grown towards the midline and fasciculated (arrowhead). Although the axon behavior is similar to wild type, the cell body positioning is irregular and more closely resembles *robo*. (C′) *robo* mutant in which the pCC axons (arrows) have crossed the midline (arrowhead) and are growing towards the descending pioneer axons. The axons are beginning to form the characteristic circular fascicles of the *robo* mutant. (A′′) Wild-type embryo in which the pioneer longitudinal tracts have continued their growth on either side of the midline but do not cross it. (B′′) *slit-UC* mutant in which the longitudinal pioneers, both ascending and descending, have chosen to cross the CNS midline rather than continue growing parallel to the CNS midline. The 1D4-positive fascicles have formed the characteristic circles of the *robo* phenotype. The positioning of cell bodies and the size of the circles formed by the pioneers continue to be irregular. (C′′) *robo* mutant in which the circular fascicles created by longitudinal pioneers recrossing the midline in every segment are visible. (D) Quantification of initial pCC midline crossing in early to mid-stage 13 embryos. The number of individual pCC axons inappropriately crossing the midline was scored for the 8 abdominal and 3 thoracic segments for individual embryos (total of 22 pCC axons; S2 Data). Genotypes are shown beneath the columns. Error bars are one standard deviation. The data were analyzed using a Tukey HSD within a one-way ANOVA. Horizontal bars indicate pairwise comparisons, and there is a statistically significant difference between *robo* mutants and *slit-UC* (*** *p* < 0.0001). (E) The crossing of pCC axons at the second commissure encountered was analyzed in the abdominal and thoracic segments of stage 14 embryos (11 segments; S3 Data). The presence of a pCC axon at or near the midline was scored as a crossing event. Genotypes are shown beneath the columns, and error bars are one standard deviation. The data were analyzed using a Tukey HSD within a one-way ANOVA. There was no significant difference between *robo* and *slit-UC* mutants. (F) Schematic representing the axon trajectories observed in *slit-UC* mutants. The pCC axons are born just posterior to a commissure (red arrow). Ninety-six percent of *robo* mutant pCC axons immediately cross this commissure, but only 30% of *slit-UC* mutant axons do. The pCC axons grow anteriorly, encountering the descending MP1 longitudinal pioneer axons. In both *robo* and *slit-UC* mutants, the pCC axon typically follows the MP1 axon to the midline. The primary difference between *robo* and *slit-UC* mutants is therefore the tendency to cross the midline at the first commissure (red arrow). The MP1 axon also inappropriately crosses the midline on encountering a commissure. For the full schematic, see S3 Fig. The underlying data are shown in S2 Data and S3 Data.

The longitudinal connectives in the fly embryo are pioneered in part by the pCC interneuron [24,25]. To understand what role Slit cleavage might be playing, we examined the behavior of the pCC axon in the *slit-UC* mutant using anti-Fas2 staining. In early stage 13 *slit-UC* embryos, the pCC axons can clearly be seen growing at different rates, supporting a role in axon growth (Fig 3B). Cell body positioning in the *slit-UC* mutant is also irregular, suggesting a role in neuronal migrations. In wild-type embryos, the pCC axon does not cross the midline, projecting ipsilaterally (Fig 3A). In contrast, in *robo1* mutants, 96% of pCC axons cross the midline in the first commissure they encounter (Fig 3C, 3C′ and 3D; S2 Data). Surprisingly, given the similarity to *robo1* mutants when stained with BP102, only 30% of pCC axons cross the midline at the first commissure in *slit-UC* mutants (Fig 3B′ and 3D; S3 Data). This difference is statistically significant (*p* < 0.0001, Tukey HSD test). The *slit-UC* allele produces a less stable Slit protein [22], which may be responsible for the pCC phenotype, but midline repulsion is clearly present. The ascending (pCC and also vMP2) and descending (MP1 and dMP2) pioneers fasciculate to form the first longitudinal tract that spans the segment boundary. In *robo1* mutants, the pioneer axons recross the midline on arriving at the next segment (stage 14), forming the characteristic circular fascicles that give the mutant its name (*roundabout*; Fig 3C′′) [7]. The same circular fascicles are seen in *slit-UC* mutants (Fig 3B′′). We scored the presence of a Fas2-positive midline-crossing fascicle at the posterior of a segment as evidence for pCC crossing the midline and found such a fascicle present in 95% of the segments examined. This frequency is statistically indistinguishable from *robo1* mutants (Fig 3E). In summary, in *slit-UC* mutants most pCC axons do not cross the midline at the first commissure they encounter but do cross at the next commissure (Fig 3F). This phenotype is quite distinct from *robo1* mutants (see the schematic in S3 Fig) and allows us to experimentally separate midline repulsion from crossing the segment boundary. The phenotype potentially reveals an inability of Slit/Robo signaling to suppress Netrin-based midline attraction, a possibility we test below. Alternative explanations include a decrease in repulsion as the embryo gets older or a need for an increase in growth cone motility to cross the segment boundary as the axon environment grows more complex.

### A Slit-N-Dependent Dscam1-Robo Complex

**Fig 4:**
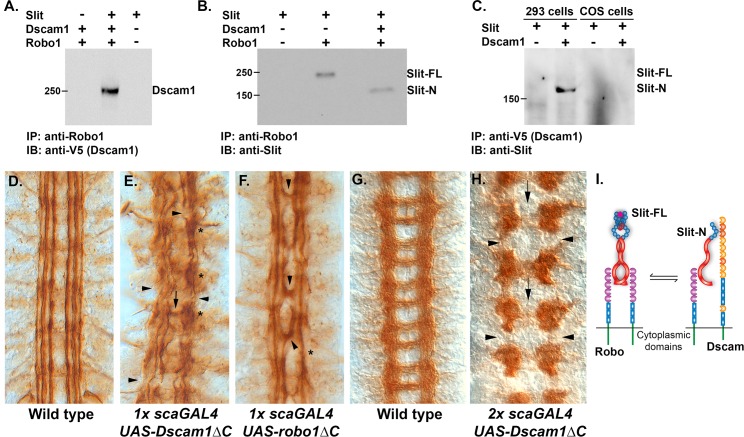
Dscam1 forms a Slit-N-dependent complex with Robo1. (A) Transfection of COS-7 and 293 cells with plasmids encoding Dscam1, Robo1, and Slit as indicated by pluses (presence of plasmid) above each lane. Immunoprecipitation with an anti-Robo1 antibody pulls down Dscam1, but only in the presence of Slit. (B) Immunoprecipitation of Robo1 alone recovers full-length Slit, but in the presence of Dscam1, Slit-N is preferentially recovered. (C) Schematic summarizing the observed interactions, suggesting that an equilibrium between Robo1 homodimers and Dscam1-Robo1 heterodimers exists. Slit-FL is depicted as dimerizing at the leucine-rich repeat 4 (LRR4) and cysteine knot domains. The stoichiometry of the Dscam1-Robo1-Slit-N complex is unknown. (D) Anti-Fasciclin 2 (MAb 1D4) staining of longitudinal fascicles. Three tracts project in an anterior-posterior direction on either side of the CNS midline, which is in the center of the picture. (E) A single copy (represented by “1x” in the figure legend) of a *Dscam1ΔC* transgene expressed pan-neurally by the *scabrous* promoter (*sca-GAL4*) reveals severe disruption of the longitudinal fascicles. In addition to the overall disorganization of the fascicles, longitudinal breaks (arrows) can also be seen, as well as clumping and stalling (asterisks). There is midline crossing at only one point (arrow). (F) A single copy of a *roboΔC* transgene expressed pan-neurally by the *scabrous* promoter reveals midline crossing of the innermost fascicle at multiple points (arrowheads). The longitudinal tracts have increased waviness and are sometimes merged or absent (asterisk), but they are generally continuous. (G) BP102 staining to reveal the wild-type CNS axon scaffold with its characteristic ladder-like pattern. (H) Multiple copies (most likely two of each, represented by “2x” in the legend) of a *Dscam1ΔC* transgene and *sca-GAL4* driver produce absence of the longitudinal connectives between segments (arrowheads) and greatly reduced midline crossing (arrows). (I) Model of Robo binding Slit-FL as a homodimer and Slit-N binding Dscam and Robo as a heterodimer; the stoichiometry of the complex is unknown at present and could involve multiple molecules. The relationship between the two complexes is shown as an equilibrium that could be altered by overexpression or removal of specific components.

As axon guidance receptors can form complexes that alter responses to ligands, we tested whether Dscam1, Robo1, and Slit could form a complex in cell culture. The receptors and Slit were expressed in 293 cells, which can proteolytically process Slit to produce Slit-N [26]. Immunoprecipitation of Robo1 can pull down Dscam1, but only in the presence of Slit, suggesting that Dscam could modify Robo signaling output (Fig 4A). When Robo1 was present alone, Slit-FL was preferentially immunoprecipitated by anti-Robo1 (Fig 4B). However, when plasmids for Robo1 and Dscam1 were present in approximately stoichiometric amounts, Slit-N was preferentially immunoprecipitated, indicating that the Dscam1-Robo1-Slit-N complex forms in preference to Robo1-Slit-FL (Fig 4B). We also tested the ability of Dscam1 to bind Slit isoforms in the absence of Robo1. We transfected 293 and COS cells with plasmids for full-length Slit and Dscam1. Immunoprecipitation of Dscam1 pulled down Slit-N, but not Slit-FL from 293 cells (Fig 4C). Our COS cell line cannot proteolytically process Slit, allowing us to test whether Dscam1 will bind to Slit-FL when Slit-N is absent. No evidence for Slit-FL binding was found, strongly suggesting that Dscam1 only binds Slit-N (Fig 4C). Expression of Robo2 increases Slit processing in vivo [27], but we found no evidence that the presence of Dscam1 influenced cleavage. Because 293 cells process Slit efficiently, the differences in processing may be best visualized in vivo, where processing is more highly regulated. This result provides a molecular explanation for Slit processing and suggests there is an equilibrium between Robo1 homodimers and the Dscam1-Robo1 complexes (Fig 4I). In flies, expression of the Netrin receptor Frazzled lacking a cytoplasmic domain (*FraΔC*) has been used to dissect the function of Fra [28,29]. If the role of Dscam1 is simply to inhibit Robo1, then a Dscam1 dominant-negative construct lacking the cytoplasmic domain (*Dscam1ΔC*) [30] would be expected to mimic expression of a similar *robo1* construct (*robo1ΔC*). Labeling of the ventral nerve cord with anti-Fasciclin 2 (Fas2) reveals three parallel longitudinal tracts on either side of the CNS midline (Fig 4D). Pan-neural expression using the *sca-GAL4* driver and a single copy (1x) of *Dscam1ΔC* is highly disruptive to the longitudinal tracts (Fig 4E). The large number of stalled axons and breaks in the tracts suggests that Dscam1 is playing a role in axon growth. In contrast, a single copy of a *robo1ΔC* transgene induces mild midline crossing as is seen for weak *robo1* mutants, although breaks in the outermost fascicle are also seen (Fig 4F) [31]. Surprisingly, when embryos expressing a single copy of *Dscam1ΔC* (1x) are stained with BP102 to reveal the CNS axon scaffold, it appears more or less like wild type, indicating that the transgenes have a disproportionately strong impact on longitudinal axon guidance. Coexpression of both *Dscam1ΔC* and *robo1ΔC* transgenes in single copies did not produce an increase in severity. The contrasting results obtained for *robo1ΔC* and *Dscam1ΔC* indicate that Dscam1 is not merely soaking up Slit-N in the embryo (in which case a *robo1*-like phenotype should be observed) but is actively signaling within the longitudinal tracts. Dscam1ΔC is likely inhibiting the activity of other receptors or complexes. Expression of multiple copies of *robo1ΔC* produces a *robo1-*like phenotype of increased midline crossing [32]. Increasing the copy number (2x) of *Dscam1ΔC* and the pan-neural driver results in disruption of both longitudinal and commissural axon guidance, and these connectives are largely missing in affected embryos (Fig 4H). The commissural axon defect confirms our earlier observations that *Dscam1* participates in growth towards the CNS midline [33]. We expect Dscam1ΔC to be binding to Slit-N and Robo1, disrupting the signaling output of the complex. As Robo1 is present in only the longitudinal portions of the CNS axon scaffold [34], Robo1/Dscam1 complexes are likely participating in longitudinal axon guidance.

### Dscam1 Has at Least Two Binding Sites for Slit-N

**Fig 5:**
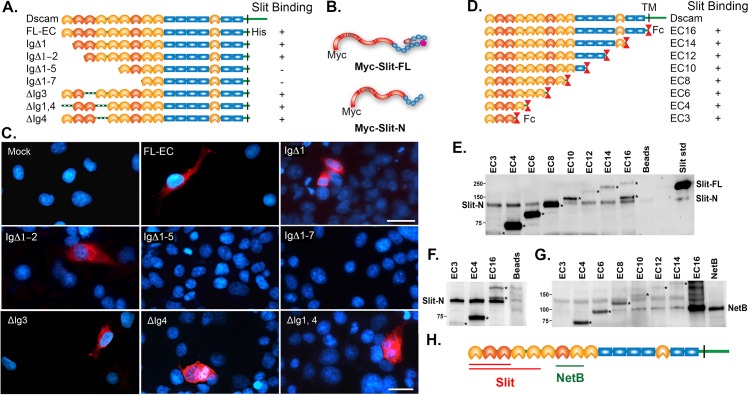
Mapping of Slit-N and Netrin-B binding sites in Dscam1. (A) Schematic representation of the Dscam1 expression constructs used. Ig domains are either orange for constant domains or red for variable domains, and fibronectin (FN) domains are blue rectangles. The naming scheme for the constructs indicates which Ig domains are deleted (Δ), while the construct with the entire ectodomain is called FL-EC. All constructs had their cytoplasmic domain replaced with a 6xHis epitope tag. Immunoblot with an anti-His antibody indicates that all constructs are expressed at their predicted sizes. (B) Schematic of N-terminally Myc epitope-tagged Slit constructs generated with leucine-rich repeats (LRRs) in red, epidermal growth factor (EGF) repeats in blue, the Laminin G domain in bright pink, and the cysteine knot in dull pink. (C) Cell overlay assays. COS cells were transfected with different Dscam1 expression constructs and tested for binding to N-terminally myc-tagged Slit-N produced by 293 cells. The relevant constructs are indicated. Binding of myc-Slit-N was detected by anti-myc (red) and counterstained with 4′,6-diamidino-2-phenylindole (DAPI) to reveal the nuclei (blue). Myc-Slit-N was found to bind FL-EC, IgΔ1, IgΔ1–2, IgΔ3, IgΔ4, and IgΔ1,4, but not IgΔ1–5 or IgΔ1–7, indicating myc-Slit-N binding in the first five Ig domains. Scale bars: 30 μm. (D) Diagram of Dscam-Fc deletion series [35]. Immunoglobulin (Ig), FN, and the transmembrane (TM) domains are indicated. The variable Ig domains are shaded black (Ig2, Ig3, and Ig7). The TM domain of full-length Dscam is replaced by an antibody constant region (Fc) domain in the deletion series. (E) Immunoprecipitation of Slit-N by Dscam-Fc fusions. Baculovirus-produced Slit-FL is loaded as a standard in the right-hand lane, and a low level of Slit-N can also be observed. Slit-FL was incubated with the EC proteins and immunoprecipitated with anti-Fc antibody, and immunoblot detection was with anti-Slit-N antibody. Slit-N was found to bind to all EC constructs tested, but not to the beads alone. Previous experiments revealed that Slit-N does not bind Fc protein alone [20]. The secondary antibody used to detect Slit-N detects the Fc fusion proteins (asterisks), including a specific degradation product for EC16. (F) Coimmunoprecipitation of Slit-N with EC proteins 3, 4, and 16 was detected by an immunoblot for Slit-N. Slit-N binds to all three constructs. Fc proteins are marked by asterisks. (G) Immunoprecipitation of NetB by Dscam-Fc fusions. Media from 293 cells expressing NetB was incubated with the EC proteins and then immunoprecipitated with anti-Fc antibody. The immunoblot was probed with anti-myc antibody to detect NetB. NetB bound to EC8 and all larger constructs. The bands migrating at the same weight as NetB in the EC6 and EC16 lanes are Dscam-Fc fusion proteins. EC16 has several degradation products when mixed with 293 cell media. We are confident that no NetB is bound in the EC6 lane. (H) Schematic summarizing the binding site data, indicating that Slit-N has at least two binding sites in the Dscam1 ectodomain and that the NetB binding site is physically distinct from the Slit binding sites.

We previously demonstrated that Slit-N binds with high affinity (22 nM) to the first four immunoglobulin (Ig) domains of Dscam1 (EC4 in Fig 5D) [20]. We attempted to further map the binding site by constructing an N-terminal deletion series of the Dscam1 gene. In all constructs, the cytoplasmic domain was replaced with a 6xHis tag, and the transmembrane domain was retained (Fig 5A). COS cell expression was confirmed by immunohistochemistry and western blot. Two Slit constructs with an N-terminal myc epitope (to avoid any steric hindrance at the cleavage site), myc-Slit-FL and myc-Slit-N (Fig 5B), produced identical bands of ~123 kD corresponding to Slit-N in National Institutes of Health (NIH) 293 cells. In cell overlay studies, Slit-N did not bind to constructs lacking the first five Ig domains or longer (IgΔ1–5 and IgΔ1–7 constructs, Fig 5C), confirming our earlier result that the binding site lies in the N-terminal ectodomain of Dscam1. Slit-N bound to the full-length Dscam1 ectodomain (EC-FL) and to constructs lacking the first (IgΔ1) and first two Ig domains (IgΔ1–2; Fig 5C). This suggested that Slit-N would bind to either Ig3 or Ig4. However, deletion of either of these domains alone did not prevent binding. We speculated that the binding sites could lie on either side of the variable domains and deleted both Ig1 and Ig4 simultaneously also without disrupting binding. These results strongly suggest that Slit-N has at least two binding sites in the first five Ig domains of Dscam1. To confirm these results, we utilized a series of constructs in which the Slit ectodomain is deleted from the carboxy-terminal end and the derivatives fused with the Fc region of human IgG (EC series, Fig 5D) [35]. We previously demonstrated that EC4 binds to Slit-N fused to alkaline phosphatase and that Slit-N does not bind Fc alone [20]. These constructs are fused to the antibody constant region, allowing immunoprecipitation. Slit proteins were produced using a baculovirus system, and in the insect cell lines used, Slit processing was observed at a low level (S4 Fig). When Slit-FL was tested for binding to the EC constructs, Slit-N was found to bind to all constructs tested, but Slit-FL did not, even though Slit-FL is present in considerable excess (Fig 5E). This implies that there is a Slit binding site in the first three Ig domains of Dscam1. We confirmed this finding with Slit-N expression alone (Fig 5F). Combined with the results in Fig 3, this suggests that Dscam1 has a strong preference for binding Slit-N. As a further control, we mapped the binding site of Netrin-B to Ig domains 7 and 8, as NetB bound to Ec8 but not Ec6 (Fig 5G). This result is consistent with those obtained for vertebrate Dscam and cNetrin-1, which placed binding in Ig domains 7–9 [36]. In summary, Slit-N binds to at least two sites in the first five Ig domains of Dscam1, with one site lying in the first three Ig domains (Fig 5H).

### *Dscam1* Modulates *slit-N* Phenotypes In Vivo

**Fig 6:**
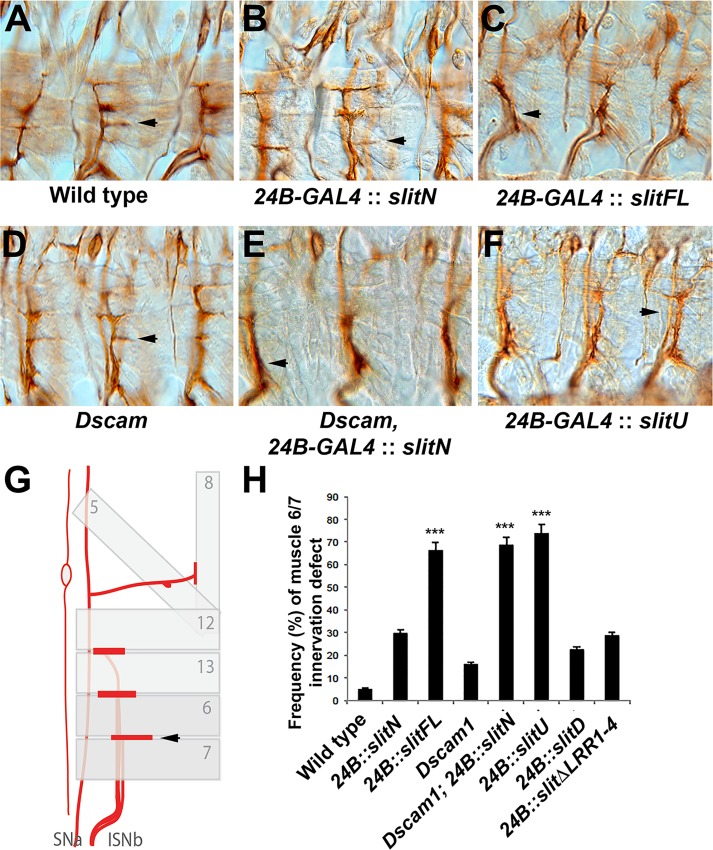
Dscam1 can modulate the differential effects of Slit-N. Motor neurons in stage 17 embryos are stained with anti-Fas2 mAb 1D4 (A–E). (A) Wild-type embryo showing a horizontal projection from the intersegmental nerve b (ISNb) nerve into the muscle 6/7 cleft (arrow). (B) Embryo overexpressing *UAS-slit-N* in muscles using the muscle-specific 24B-GAL4 driver. In this example, muscle 6/7 innervation appeared slightly weaker but was still present (arrow). The adjacent segments have robust innervation. (C) Muscle expression of *UAS-Slit-FL* repels the ISNb so that innervation does not occur (arrow). (D) *Dscam1* mutants have a low level of innervation defects at muscle 6/7. This example shows a normal innervation (arrow), while the segment to the right has a smaller horizontal branch. (E) Muscle overexpression of *UAS-Slit-N* in a *Dscam1* mutant leads to a strong repulsion phenotype and failure to innervate muscle 6/7 (arrow). (F) Overexpression of *UAS-slit-U* in muscles strongly repels motor neurons. (G) The schematic demonstrates how the ISNb nerve assayed runs under the body wall muscles to innervate the cleft between muscles 6 and 7 (arrow). (H) Quantification of the muscle 6/7 innervation defects expressing as a percentage compiled from ten embryos (S4 Data). Transgenic overexpression of *UAS-Slit-N* in a *Dscam1* mutant background or over-expression of *UAS-Slit-FL* or *UAS-Slit-U* (uncleavable) is statistically different from wild-type controls, *Dscam1* mutants, and *UAS-Slit-N* overexpression (*** *p* < 0.001 in a Tukey HSD test within a one-way ANOVA). The underlying data are shown in S4 Data.

The preferential binding of Slit-N to Dscam1 led us to identify an assay system in which we could observe differential effects of Slit-FL and Slit-N in vivo. Motor neurons express *robo1* [37] and *Dscam1* (S5 Fig). *Dscam1* mutants show motor neuron innervation defects, suggesting *Dscam1* is active in the process. Dscam1 is also seen at a low level at muscle attachment sites (S5 Fig), so Dscam1 may be present on the muscles themselves. Muscles do not normally express *slit* as they respond to Slit at muscle attachment sites [22,38]. Muscle overexpression of *UAS-slit-FL* in muscles has been previously shown to repel innervating motor neurons [39]. We found that overexpression of *UAS-slit-N* only repelled 30% of motor neurons innervating the muscle 6/7 cleft, whereas *UAS-slit-FL* repelled 70% of axons (Fig 6B and 6C). Expression of an uncleavable *slit* transgene (*UAS-slit-U*) repelled 74% of motor neurons (Fig 6F), the same level as *UAS-slit-FL*. Removal of *Dscam1* activity increased the repulsive effect of *UAS-slit-N* to a level that is statistically equivalent to that of *UAS-slit-FL*, but muscle innervation was different from overexpression of *UAS-slit-N* alone or the failure of innervation seen in *Dscam1* homozygotes (*p* < 0.001, Tukey HSD test, Fig 6H). As muscles do not normally express slit, and Dscam1 may be present on the muscles at a low level, these results must be interpreted with caution. As Dscam1 modulates the response of motor neurons to ectopically expressed Slit-N, it may be acting as a receptor in vivo. However, loss of *Dscam1* may allow the expression or activation of an alternative receptor that responds directly to Slit-N. Dscam1-Dscam1 interactions between the motor neuron and muscle could also be mediating a repulsive effect. In the absence of *Dscam1*, Slit-N appears to be as repulsive as Slit-FL or Slit-U. Motor neuron growth cones actively integrate both positive and negative cues [40]. Slit-N may have both a repulsive activity and a second activity such as promoting axon outgrowth that may have conflicting effects on motor neurons.

### Embryonic Dscam1-Slit Signaling Requires the Robo Binding Site in Slit

**Fig 7:**
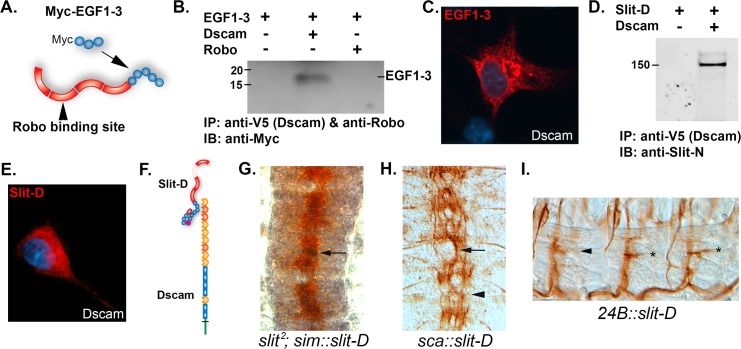
Mapping of the Dscam1 binding site on Slit. (A) Diagram of Slit-N protein showing the LRRs in red and the EGF repeats as blue circles. The three EGF repeats used to make the N-terminal Myc-tagged construct (Myc-EGF1-3) are shown. The Robo binding site of LRR2 is indicated. (B) Immunoprecipitation of Myc-EGF1-3 by Dscam but not Robo. HEK 293 cells were transfected with the Myc-EGF1-3 plasmid, and COS-7 cells were transfected with 3 ug Dscam and Robo individually, as indicated above the immunoblot. Dscam1 was immunoprecipitated using a C-terminal V5 epitope tag, and Robo was immunoprecipitated using the 13C9 mAB. The immunoblot was probed with anti-Myc to detect the EGF1-3 protein. A band is observed only in the Dscam1 lane. (C) Cell overlay assay. COS-7 cells were transfected with Dscam1 plasmid and incubated with media from 293 cells transfected with the Myc-EGF1-3 construct. Binding of EGF1-3 was detected by anti-Myc immunofluorescence (red), and the cell nuclei were counterstained with DAPI (blue). (D) A Slit derivative lacking the Robo binding site (Slit-D) physically associates with Dscam1 in an immunoprecipitation assay. HEK 293 cells were transfected with *slit-D* and *Dscam1* constructs (+ and − above the blot) and immunoprecipitated with anti-V5 antibody to immunoprecipitate Dscam1 via an epitope tag. The blot was probed with anti-Slit-N antibody. A 150 kD band corresponding to full-length (unprocessed) Slit-D immunoprecipitates in the presence of Dscam1, suggesting that inhibition of binding by the C-terminal domain of Slit is relieved by removal of the N-terminal LRR2 domain. (E) Cell overlay assay in which media containing Slit-D were incubated with COS-7 cells expressing Dscam1. Binding of Slit-D to the cells was detected with the anti-Slit (C555) antibody as revealed by red immunofluorescence. (F) Diagram summarizing the mapping results, showing Dscam1 binding Slit-D via the EGF repeats and that binding is unaffected by the absence of the LRR2 Robo binding site. (G) Midline expression of *slit-D* using the *sim-GAL4* driver fails to rescue the *slit* mutant phenotype. BP102 staining (brown) shows the characteristic collapse of the CNS axon scaffold onto the midline (compare to Fig 1B). Expression of *slit-D* (black, arrow) is predominantly in the midline cells lying underneath the axons so it is only partially visible. (H) Pan-neural expression of *slit-D* using the *sca-GAL4* driver disrupts longitudinal axon guidance. Staining of the longitudinal fascicles using MAb 1D4 (brown) reveals disruption of the six parallel fascicles (compare to Fig 1G) so that a large fascicle meanders along the midline. (I) Muscles expressing *slit-D* and stained for motor neurons with MAb 1D4 showing no effect (asterisks) or a weak effect (arrowhead) on muscle 6/7 innervation. Quantification (Fig 6H) reveals a weak inhibition of innervation. The underlying data are shown in S4 Data.

Previous genetic analysis of *slit* suggested that deletion of the epidermal growth factor (EGF) domains enhances the repulsive function of *slit* [41]. Expression of a *slit* construct lacking the EGF domains also disrupted longitudinal axon guidance, suggesting the EGF domains might be the Dscam1 binding site [41]. We expressed EGF domains 1–3 in cell culture and were able to immunoprecipitate the EGF domains with anti-Dscam1, but not with anti-Robo1 (Fig 7A and 7B). We confirmed the interaction using a cell overlay assay (Fig 7C). To test the involvement of Robo in Slit-N signaling, a *slit* construct with the Robo binding site of leucine-rich repeat 2 (LRR2) deleted was generated (Slit-D). Slit-D was found to still be capable of binding Dscam1 in an immunoprecipitation assay (Fig 7D). Interestingly, the bound Slit-D protein is the uncleaved form, indicating that Slit-D can bind to Dscam1 in the absence of proteolytic processing. Slit-D also bound Dscam1 in a cell overlay assay (Fig 7E), confirming that the Dscam1 binding site is distinct from the Robo binding site (schematic in Fig 7F). We expressed *slit-D* at the CNS midline in a *slit* mutant, and as expected, *slit-D* was unable to rescue the collapse of axons onto the CNS midline (Fig 7G). However, pan-neural expression of *slit-D* disrupted the guidance of all longitudinal tracts and caused the appearance of a large fascicle wandering the midline that was reminiscent of *slit-U*, *Dscam1 robo1*, and *lola* mutants (Fig 7H). Slit-D may be acting as a dominant-negative binding to Dscam receptors and/or endogenous Slit. We expressed *slit-D* in muscles to test whether removal of the Robo binding site could unmask a positive activity of Dscam1-Slit signaling. However, overexpression of *slit-D* actually inhibited innervation at muscle 6/7 to the same extent as *Dscam1* mutants, suggesting that Slit-D may act as a dominant negative to inhibit Dscam1 function (Figs 6H and 7I). Similar results were obtained for a construct lacking all the leucine-rich repeat (LRR) domains (ΔL1–L4; Fig 6H), which has no effect when expressed in the CNS [41]. Deletion of the Robo binding site therefore appears to inactivate Slit’s biological activity even though Slit-D remains capable of binding Dscam1, suggesting that in the embryo Dscam1-Slit signaling is Robo dependent.

### Lateral Expression of *slit-N* Partially Suppresses *slit* Mutant Phenotypes

**Fig 8:**
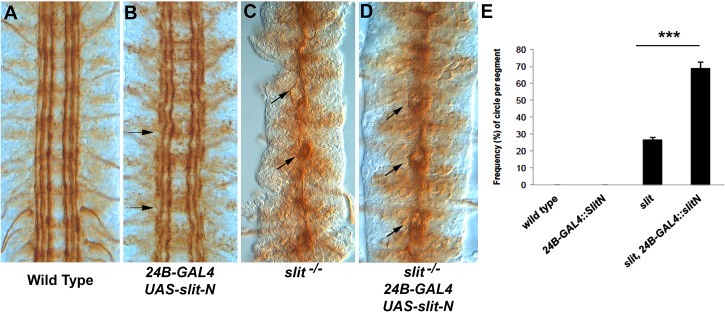
Partial suppression of the *slit* CNS phenotype by *slit-N* expression. The longitudinal tracts of stage 17 embryos were stained with anti-Fas2. (A) Stage 17 wild-type control embryo. (B) Expression of *slit-N* lateral to the CNS using the 24B muscle-specific promoter. Some waviness of the tracts and occasional breaks (arrows) are seen, suggesting that the ectopic *slit-N* is disrupting normal guidance. (C) A *slit* mutant displaying the characteristic collapse of the tracts onto the CNS midline. An occasional separation of the fascicles, which appears as a small circle, can be seen (arrow), as opposed to a failure of motor neuron bundles to leave the CNS (arrowhead). The motor neurons are easily distinguished by their position and the absence of the motor nerve root in that segment and were not counted in analysis of phenotypes. (D) Expression of *slit-N* in a *slit* mutant induces frequent separation of the fascicles at the CNS midline, appearing as small circles (arrows). These circles are slightly larger and more frequent than those seen in the *slit* mutant alone. (E) Quantification of the number of circles visible in the abdominal and thoracic segments expressed as percentages for the genotypes indicated (S5 Data). We have never observed the circles in wild type or in embryos expressing *slit-N* by *24B-GAL4*. A statistically significant difference between *slit* and *slit* with lateral expression of *slit-N* was determined using a Tukey HSD within a one-way ANOVA, ***p < 0.001. The underlying data are shown in S5 Data.

The pCC axon behavior in *slit-UC* is distinct from that in *robo1* mutants. A possible explanation is that cleavage of Slit allows Slit-N to suppress attraction to the CNS midline [14]. If this model is true, then it should be possible to suppress the *slit* mutant phenotype by expressing *slit-N* at places other than the midline (midline expression would not allow us to distinguish between repulsion and suppression of attraction). In *slit* mutants, the longitudinal fascicles are attracted to and collapse onto the CNS midline, with separation between the fascicles limited to occasional very small circles (Fig 8C), similar to those seen in *robo1* mutants but much smaller. We used the *24B-GAL4* muscle driver to express *slit-N* lateral to the CNS, as muscle precursors are present to the sides of the developing CNS (a subset lie on top of the CNS as well). Expression of *slit-N* in *slit* mutants induced separation of fascicles such that the fascicles formed small circles (Fig 8D). Quantification of the phenotype revealed it to be statistically significant (Fig 8E). The CNS phenotype of homozygous *slit* mutants is very strong and challenging to suppress, and most experiments typically use *slit/+* heterozygotes to detect effects. *Netrin* mutants can weakly suppress *slit* homozygotes [19], and our results with ectopic Slit-N are similar in strength of suppression.

### *NetrinA*,*B Dscam1* Double Mutants Have Increased CNS Axon Defects

**Fig 9:**
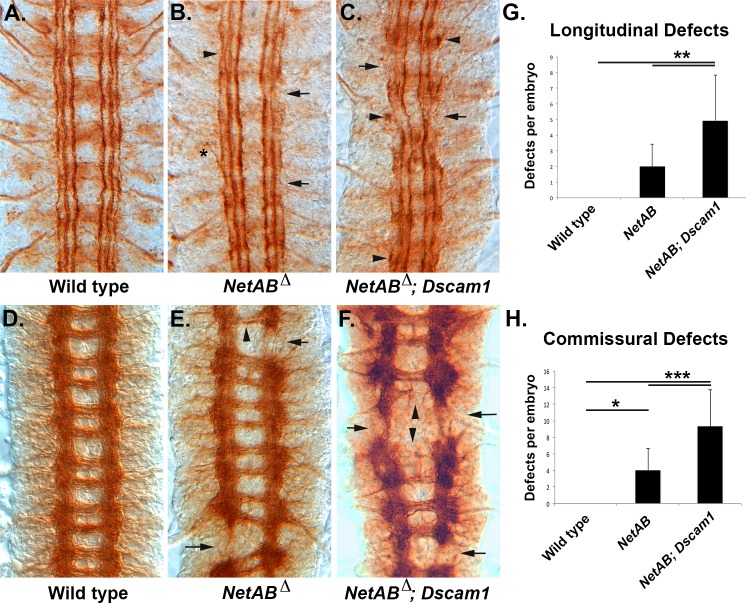
*NetrinA*,*B Dscam1* double mutants have enhanced axon defects. (A–C) Stage 17 embryos stained with 1D4 to visualize the longitudinal tracts. (D–F) Stage 16 embryos stained with BP102 to visualize the CNS axon scaffold. (A) Wild-type embryo showing the six longitudinal tracts running parallel to the CNS midline. (B) Embryo lacking both *Netrin A* and *B* (*NetAB*) genes. Occasional gaps in the outermost fascicle are visible (arrows), as well as defasciculation of tracts (arrowhead), and exit of axons from the CNS (asterisk). (C) Embryo doubly mutant for *NetAB* and *Dscam1*. There is an increase in the number of outermost fascicle breaks, the medial fascicle is also reduced or absent (arrows), and stalled axons can be seen (arrowheads). (D) Wild-type embryo with the characteristic ladder-like pattern of the CNS axon scaffold. (E) *NetAB* mutant embryo with breaks in the longitudinal connectives (arrows) and commissures of reduced thickness (arrowhead). (F) *NetAB; Dscam1* double mutant embryo with breaks in the longitudinal connectives (arrows) and greatly reduced or absent commissures (arrowheads). The increased BP102 signal in this embryo is due to an increase in the length of staining, not the genotype. (G) Longitudinal connectives stained with BP102 were scored in the abdominal and thoracic segments of stage 16–17 embryos (S6 Data). A defect was scored if the tract was obviously thinner or absent. The average value is shown, and error bars are one standard deviation. The data were analyzed by Tukey HSD within a one-way ANOVA, and *NetAB; Dscam1* embryos were found to be significantly different from *NetAB* embryos (**, *p* = 0.0013). (H) BP102-stained commissures were scored for absence or significant reduction in thickness in stage 16–17 embryos in 11 segments per embryo (S7 Data). The average value for each genotype is shown, and error bars are one standard deviation. Wild type was found to be significantly different from *NetAB* (*, *p* = 0.0272) and *NetAB; Dscam1* (***, *p* = 0.0001). *NetAB; Dscam1* was significantly different from *NetAB* (***, *p* = 0.0003). All statistics were Tukey HSD. The underlying data are shown in S6 Data and S7 Data.

**Fig 10:**
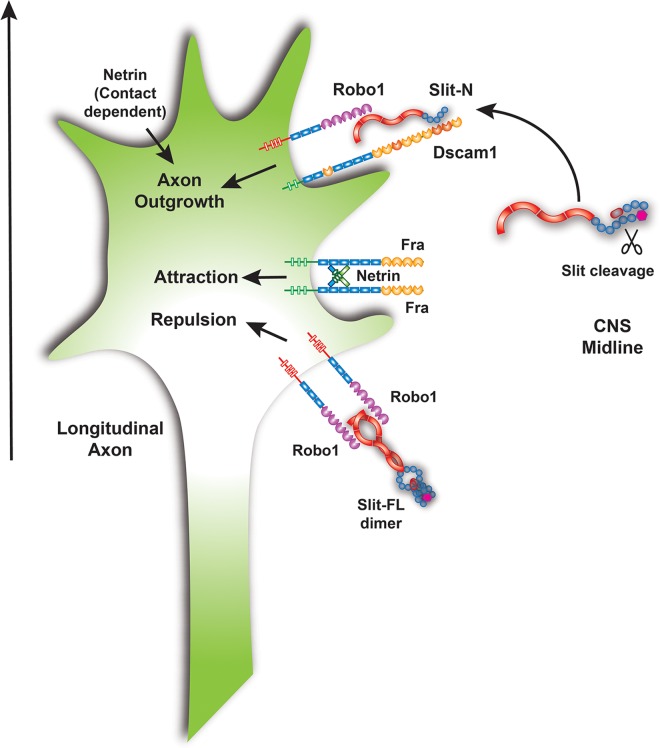
Model for the function of Dscam1-Robo1-Slit-N signaling in longitudinal axon guidance. Slit is expressed by midline glia at the CNS midline and likely processed after secretion. Slit-N can form a complex with Dscam1 and Robo1 to promote longitudinal axon growth. The stoichiometry of the Slit-N-Dscam1-Robo1 complex is unknown, but Slit-N dimerization could potentially recruit two Robo1 and two Dscam1 molecules to the complex. Full-length Slit is shown binding as a dimer to Robo receptors, promoting repulsion. Netrin is shown binding to Frazzled receptors as a dimer based on vertebrate data for DCC/Neogenin and promotes attraction to the CNS midline. Vertebrate data suggest that integration of conflicting attractive and repulsive cues may also promote axon growth. Netrin promotes longitudinal growth via an unknown receptor and a contact-dependent mechanism. The combination of signals received by the growth cone serve to promote axon growth parallel to the midline (arrow).

If Dscam1 is acting to suppress attraction to midline Netrins, then *NetAB* mutants should partially suppress the *Dscam1* longitudinal phenotype. *NetAB* mutants have mature longitudinal tracts that, although largely intact, often show breaks in the outermost fascicle and defasciculation (Fig 9B). When combined with a *Dscam1* mutation, the longitudinal tracts show much greater disruption of the outermost fascicles and occasionally the medial fascicle, as well as a lot of axon stalling (Fig 9C). When stained with BP102 to visualize the entire CNS axon scaffold, *NetAB* mutants display missing longitudinal connectives (Fig 9E; Table S2 in [42]). When stained with BP102, *NetAB; Dscam1* double mutants have dramatic disruptions of the longitudinal connectives and commissures (Fig 9F). The *NetAB* phenotype is quite variable, and this is reflected in a wide range of phenotypes observed in *NetAB; Dscam1* double mutants (S6 Fig). We quantified these defects and found statistically significant difference between *NetAB* and *NetAB; Dscam1* for both commissures (*p* < 0.0005, Tukey HSD; S6 Data) and longitudinal tracts (*p* < 0.005, Tukey HSD; S7 Data). This result makes it extremely unlikely that Dscam1 is required to suppress attraction to midline-derived Netrins in longitudinal axons. *Dscam1* enhances both the midline crossing and longitudinal connective defects of *NetAB*, and we favor a model in which an axon growth-promoting activity of Dscam1 is responsible (Fig 10 and below).

## Discussion

In this study, we determined that one function of Slit cleavage is to allow Slit-N binding to Dscam1 and to promote the formation of a complex between Dscam1 and Robo1 (Fig 10). All three components of this complex are required for pioneer axon growth across segment boundaries. Several lines of evidence suggest that the complex transduces an axon growth-promoting signal. These findings establish that one function of Slit cleavage in vivo is to function in longitudinal axon guidance.

### Longitudinal Axon Guidance

Pioneer longitudinal axons need to traverse segment boundaries. Previous work has established that crossing the boundary requires a combination of pathway cues and growth cone motility for axons to connect adjacent segments [12]. Longitudinal glia and a mesh of neuronal filopodia provide signals to guide pioneers to and across the segment boundary [12,43]. Netrins guide the positioning of the longitudinal glia [44], are also trapped by the Fra receptor on the neuronal mesh, and are presented to the navigating pioneers during their initial trajectories [13]. In *NetAB* and *fra* mutants, the dMP2 descending pioneer axon makes guidance errors, and this may underlie the defects seen in the longitudinal connectives at the end of embryogenesis [45–47]. Our finding that the *NetAB; Dscam1* double mutant strongly disrupts longitudinal connectives confirms that these genes play important roles in this process. It is noteworthy that the *lola* transcription factor regulates expression of *fra*, as well as *robos1-3*, *slit*, and *Dscam1*, potentially explaining why *lola* has such a profound longitudinal axon phenotype [9].

The significance of the segment boundary as a barrier to axon growth is well established in the homologous grasshopper CNS, in which the growth cones of the ascending and descending longitudinal pioneers pause at the segment boundary for 5 h until selective fasciculation between the pioneers is established. Ablation studies revealed that selective fasciculation is absolutely critical for longitudinal growth [48]. In the fly CNS, the physical distances are significantly smaller, and fasciculation may play a lesser role [25]. For example, filopodia from the pCC pioneer can contact glia on the other side of the segment boundary before the arrival of dMP2/MP1 axons [43]. Mutants in *Fas2* that prevent fasciculation of the pioneers do not disrupt pioneer guidance [49]. Nevertheless, the descending MP1 and dMP2 growth cones pause at the segment boundary and wait for the ascending pCC and vMP2 axons to arrive [25]. In addition, mutants in the adhesion molecule *N-cadherin* (*CadN*) can display breaks in the MP1 pathway that are suggestive of axon stalling [50]. The mature *CadN* phenotype closely resembles that of *Dscam1* mutants, and our observation that *NetAB* mutants have fasciculation defects suggests that both *Dscam1* and *NetAB* are required in axons that follow the pioneers. It was predicted that longitudinal axon phenotypes might be more subtle in the fly, as the shorter distances would allow guidance defects to self-correct over time through the use of parallel pathways [25]. This may explain why pioneer defects in mutants such as *CadN* and *NetAB* only result in subtle defects in outermost fascicle of the mature Fas2 pattern. Detecting strong defects in pioneer axons will likely require knockdown of parallel pathways at the same time, and we are actively testing this hypothesis with *Dscam1*.

### Growth Cone Motility

In flies, growth cone motility appears to be another critical requirement for crossing the segment boundary. Temperature-shifted *Notch* mutants display strong segment crossing defects. Activation of noncanonical Notch signaling in the pioneer axons is sufficient to rescue the mutant defects because of an increase in growth cone motility. Notch signaling promotes the formation of filopodia, which may allow the growth cone to sample growth-promoting cues on the other side of the segment boundary [12]. In vertebrates, the simultaneous and conflicting actions of midline attractants and repellents on longitudinal growth cones have been proposed to drive forward growth parallel to the midline, in the so-called “push-pull” model [5]. Interestingly, part of the chemorepellent activity of Slit is the ability to increase axon outgrowth of cultured axons [5,51]. Expression of Slit and Netrin in the spinal cord is continuous [52,53], but the segmented nature of the fly ventral nerve cord means that expression occurs at discrete locations. It may therefore be necessary to increase motility at the segment boundary to ensure crossing. We propose that this is the function of the Dscam1-Slit-N-Robo1 complex, and we discuss this in detail below. It is important to note that alternative mechanisms are also possible. Slit-N bound to axons after diffusing from the midline could be acting as a contact-dependent attractant, either neutralizing Dscam1-mediated homophilic repulsion between axons [54] or promoting attachment between the longitudinal axons and their substrate as has been proposed for Slit-N in muscles [27]. The complex could be trapping Slit-N for presentation to additional receptors as has been proposed for Robo1 and Syndecan [37,55]. Slit-N signaling could also modulate fasciculation via regulation of cadherins [56].

### The Role of the Dscam1-Slit-N-Robo1 Complex in Longitudinal Axon Guidance

While most prior studies on fly Dscam1 have focused on dendrite inhibition, Dscam1 clearly also has a positive role in axon outgrowth, as Dscam1 is required for the growth of presynaptic arbors and overexpression increases the size, branch length, and branching of arbors [57,58]. Differences in elongation rates for longitudinal pioneers have been described in *robo1* mutants [14]. Our work suggests that *Dscam1∆C* disrupts axon growth in both commissures and longitudinal tracts (Fig 4E and 4H). The *Dscam1 robo1* and *slit-UC* mutants have significant breaks in the longitudinal fascicles suggestive of stalling at the segment boundary (Fig 1J and 1K), and examination of the pCC growth cones in the *slit-UC* mutant reveals different rates of outgrowth (Fig 3B). Pan-neural expression of the *slit-D* construct also causes longitudinal breaks (Fig 7H). Finally, lateral expression of *Slit-N* causes a modest promotion of growth away from the midline in *slit* mutants (Fig 8). Taken together, these results suggest that Dscam1, Robo1, and Slit-N all play a positive role in axon outgrowth, especially in longitudinal axons. Lack of a growth-promoting signal in *slit-UC* or *Dscam1 robo1* mutants would prevent pioneer axons from crossing the segment boundary.

In previous studies, the longitudinal pioneers have been observed to stall, exit the CNS, or grow in the opposite direction when guidance is disrupted [12,13,48]. The *slit-UC* allele used in our work encodes a protein with decreased stability [22], so it is likely to be acting as a hypomorphic allele with reduced repulsive function. The reduction in repulsion in *slit-UC* or *Dscam1 robo1* mutants allows axons that do not stall to inappropriately enter the midline, resulting in a large central fascicle that wanders back and forth across the midline (Fig 1J and 1K). A priority for us is to construct additional *slit-UC* alleles with hopefully greater protein stability through CRISPR-Cas9 based approaches. This may allow clearer visualization of axon growth phenotypes.

Our interpretation of the role of the Dscam1-Robo1-Slit-N complex relies heavily on prior studies of Dscam1 and limited observations of growth rate differences in the *slit-UC* mutant. Our observations have been complicated because of the components of the complex participating in additional processes, with *Dscam1* being required for midline crossing and *robo1* and *slit-UC* being required for midline repulsion. Crossing the midline is an alternative path for a longitudinal axon to take if it fails to cross the segment boundary. We hope to uncover stronger, more definitive phenotypes through construction of double mutants with other longitudinal components such as *CadN*. An alternative approach will be to visualize filopodial dynamics in pioneer growth cones in living embryos as a measure of axon outgrowth. Again, this may be complicated by the nature of the complex components. For example, the repellent function of Slit/Robo1 normally limits the number of filopodia, and loss of *robo1* produces a more highly branched growth cone with longer filopodia [59]. However, it was recently shown that Slit protein initially stimulates filopodial extension towards sources of Slit [60], and subsequent endocytosis of Robo-Slit complexes may then lead to filopodial retraction and growth cone repulsion [61]. Dscam is likely to influence filopodial dynamics as well [62], maybe as simply as by modifying Robo1 activity. Proving an effect of the complex on growth will require genetic backgrounds and tools that clearly separate the different activities of the components. Such analysis may be more definitively carried out in cultured neuron systems [60].

### Lack of Suppression of Netrin-Mediated Attraction

The primary role of Robo/Slit signaling is repulsion from the CNS midline. Ectopic *slit* expression in lateral neurons can guide dMP2 axons, revealing a second function for Slit [14]. As the Robo1-positive dMP2 growth cones grow right over the source of ectopic Slit and are not repelled, this indicates that Slit is not acting as a directional cue. The Slit activity has been proposed to suppress Netrin-mediated attraction to the midline, thereby ensuring that longitudinal axons continue projecting parallel to the midline [14]. The Dscam1-Slit-N-Robo1 complex is an obvious candidate to mediate this activity, but our results suggest that this is not the case. If the Netrin and Dscam pathways were interacting, one would expect a decrease in longitudinal defects. However, the *NetAB; Dscam1* double mutants have widespread disruption of longitudinal tracts and also commissures (Fig 9, S6 Fig). This result demonstrates that Dscam1-based signaling is operating in parallel to Netrins in both outgrowth processes.

### Implications for Dscam Function in Axon Guidance

Dscams are evolutionarily conserved Netrin receptors, yet they do not play a role in Netrin-mediated axon guidance in the vertebrate spinal cord [33,36,63,64]. The emerging picture that Dscam1 promotes axon growth suggests a solution of this paradox. Neurotrophic factors are among the key extracellular signals that promote axon growth [65]. When tested in vitro, high concentrations of trophic factors can orient axon outgrowth, but they have minor effects on axon guidance in vivo [66]. If Dscams are transducing growth-promoting signals, this would explain why strong Dscam effects on axon guidance in vivo are only observed in conjunction with mutants in bona fide guidance pathways. For example, Netrins A and B are required for attraction to the CNS midline and for longitudinal axon guidance. When a *NetAB* mutant is combined with a *Dscam1* mutant, both commissures and longitudinals are affected. When *Dscam1* is combined with a *robo1* mutation, only the longitudinals are disrupted. *Dscam1* is therefore enhancing defects specific to the axon guidance pathway being tested.

### Summary

Our data support a model in which the Dscam1-Robo1-Slit-N complex transduces an axon growth-promoting signal that enables the growth of longitudinal axon pioneers across the segment boundary.

## Materials and Methods

### Reagents and Chemicals

FBS was from Atlanta Biologicals (Flowery Branch, Georgia). SF-900 II (1X) and (1.3X) medium, Express Five, SFM, Grace's Insect Medium (unsupplemented), Bac-to-Bac HBM TOPO Secreted Expression kit, High Five cells, pSecTag/FRT/V5-His TOPO TA Expression Kit, DMEM, TrypLE Express, lipofectamine 2000/3000, Cellfectin II Reagent Dynabeads, blasticidin, and Opti-MEM were purchased from Life Technologies (Grand Island, New York). The Sf9 cells, 293 cells, and COS-7 cells were gifts from C. Tittiger (Department of Biochemistry, University of Nevada, Reno, Nevada), C. Singer (Department of Pharmacology, UNR), and G. Mastick (Department of Biology, UNR), respectively. COS-7 cells were also purchased from the American Type Culture Collection. Dscam1 pIB/Fc constructs were a generous gift from W. Wojtowicz [35]. Agarose was from Bio-Express (Kaysville, Utah). Phenylmethylsulfonyl fluoride (PMSF) and Protease Inhibitor Cocktail were from Sigma-Aldrich (St. Louis, Missouri). Batimastat was from TOCRIS (San Diego, California). Gammabind Protein G Sepharose was from GE Healthcare (Pittsburgh, Pennsylvania). Heparin was purchased from Fisher Scientific (Hanover Park, Illinois). QIAprep Spin Miniprep Kit was purchased from Qiagen (Valencia, California). All oligonucleotides were from Integrated DNA Technologies (Coralville, Iowa).

### Recombinant Baculovirus Production

To generate recombinant baculovirus-full-length Slit and the N-terminal Slit cleavage fragment, pcDNA-Slit [26] was used as template and amplified by PCR (Table 1). PCR products were gel-purified and cloned into the pFastBac/HBM-TOPO vector, an entry clone vector. The recombinant plasmids were fully sequenced, purified, and transformed into DH10Bac, which contains a baculovirus shuttle vector (bacmid) and a helper plasmid to facilitate the generation of a recombinant bacmid by white-blue colony screening. The correct recombinant bacmid DNA was transfected into Sf9 cells to produce recombinant baculovirus particle. The baculoviral stock was used to infect High Five cells to express recombinant protein. All the steps were performed as described by the Bac-to-Bac HBM TOPO Secreted Expression kit manual. All the recombinant plasmids were confirmed by sequencing with primer walking at the Nevada Genomics Center (UNR), and the sequences were analyzed using Vector NTI Advance 9 software (Invitrogen).

**Table 1:** PCR primers used for DNA constructs.

Construct	Forward Primer	Reverse Primer	Template
*robo1ΔC*	GAGTGGTGAATTCAACAGCACCAAAACCACAAAATGCATCCC	CTTAAGTGACTCTAGACCTTGGTCATTTGATGC	pBS-Robo [34]
FL-EC	GGTAGTCAGACCCTAGCTG	ATCGTAGTACACATCCTTC	pcDNA-Dscam1 [33]
IgΔ1	GTGCGAGCCGTGGTTGCC	ATCGTAGTACACATCCTTC	pcDNA-Dscam1 [33]
IgΔ1–2	AAAGGACGATTGGTCATC	ATCGTAGTACACATCCTTC	pcDNA-Dscam1 [33]
IgΔ1–5	AATGTATATGGACTGCCC	ATCGTAGTACACATCCTTC	pcDNA-Dscam1 [33]
IgΔ1–7	AACGTCTACGTACCTCCC	ATCGTAGTACACATCCTTC	pcDNA-Dscam1 [33]
ΔIg3	CTATCCGCTAAGATCGATCCCCCCACCCAGACA	GATCTTAGCGGATAGTACAGCACTGCTTATAGG	pcDNA-Dscam1 [33]
ΔIg4	AAGCTCGGAGGCCGTTTCGATCCCCCCGTCATC	ACGGCCTCCGAGCTTGACGGTAAGCACAGTCTC	pcDNA-Dscam1 [33]
ΔIg1–4	AAGCTCGGAGGCCGTTTCGATCCCCCCGTCATC	ACGGCCTCCGAGCTTGACGGTAAGCACAGTCTC	IgΔ1
Myc-Slit-FL	GAACAAAAACTTATTTCTGAAGAAGATCTGGGTGGACTGGGGTCAGTG	TCAGTAGCATTTCTTGGTGC	pcDNA-Slit [26]
Myc-Slit-N	GAACAAAAACTTATTTCTGAAGAAGATCTGGGTGGACTGGGGTCAGTG	TCATGGATACATCATCGAG	pcDNA-slit [26]
Bac-Slit-FL	GAACCGTATTCCGGCGGATTC	GTAGCATTTCTTGGTGCATCC	pcDNA-slit [26]
Bac-Slit-N	GAACCGTATTCCGGCGGATTC	CTGTGGATACATCATCGAG	pcDNA-slit [26]
Myc-EGF1-3	GAACAAAAACTTATTTCTGAAGAAGATCTGGGCGGCGGCCCGTGCCAGAATCAAGCGCAG	TTTGGTGTCGCAGAACTCGCCACT	pcDNA-slit [26]

### Recombinant Baculoviral Proteins

Protocols for growth and maintenance of Sf9 cells and High Five cells, recombinant baculovirus construction, and heterologous expression using the Bac-to-Bac HBM Expression Kit were as described by Life Technologies. Briefly, an LR recombination reaction between each entry recombinant clone and a baculovirus shutter vector in DH10Bac cells produced recombinant baculovirus clones that were transfected separately into Sf9 cells for recombinant virus production. High titer P3 viral stocks for each construct were produced by successive 72-h amplifications of the initial and P2 stocks. Approximate viral titers were determined by a plaque assay. The viral stocks were used to infect High Five cells grown to a density of 1.0 x 10^6^ cells/ml in a disposable shaking flask (Bio-Express, Kaysville, Utah). For the expression of Slit-FL and Slit-N, High Five cells were infected with recombinant baculovirus at multiplicities of infection (MOIs, pfu/cell) ranging from 1 to 20. Media containing recombinant proteins were harvested at days 1, 2, 3, and 4 post infection (PI) with various MOI. Western blotting was used to determine the best expression time and MOI. All combinations of conditions for High Five cells and Sf9 cells were cultured at 27°C with shaking flasks.

### Recombinant Protein Detection

Cells infected with 6xHis-tagged Slit-N and Slit-FL recombinant baculovirus were harvested at 1–4 d PI with various MOI. The cell pellets were homogenized, and culture medium was collected. Noninfected cells were prepared similarly as a negative control. Protein production was confirmed by western blotting using 1:1000 rabbit anti-his primary antibody from Abcam ab9108 (Cambridge, Massachusetts) for Slit-N and Slit-FL, 1:250 goat anti-rabbit lgG HRP secondary antibody (Jackson Labs), and SuperSignal West Pico Chemiluminescent Substrate (Pierce, Rockford, Illinois). Images were collected with FOTO/Analyst ImageTech (FOTODYNE, Hartland, Wisconsin).

### Antibody Production

GenScript (Piscataway, New Jersey) was contracted to produce a rabbit anti-SlitN polyclonal antibody based on the antigenic peptide (CQLGENKIKEISNKM) designed and selected by the contracted company. The synthesized peptide was injected into the two rabbits separately. Four immunizations per rabbit were performed. Serum was collected, and the antibody was purified using peptide-conjugated resin. The quality of the antibody was examined by ELISA. The antibody was used to confirm recombinant protein produced in infected insect cells and in mammalian cells.

### Generation of Stable Insect Cell Lines

Eight Dscam-Fc expression constructs in the pIB/V5-His vector [35] were transfected into either Sf9 cells or High Five cells with Cellfectin II reagent. Forty-eight hours post transfection, the medium was replaced with fresh culture medium. Cells were split at 1:5 and cultured overnight with culture medium when they were confluent, followed by removal of the medium and replacement with selective medium containing 50 μg/ml Blasticidin. The selective medium was replaced every 3 to 4 d until foci were forming, and the mock cells had completely died. Resistant cells were expanded into flasks for frozen stocks with selective reagent. Blasticidin was used at a concentration of 10 μg/ml for Sf9 cells and 20 μg/ml for High Five cells for maintenance after selection. Medium without blasticidin was used when splitting cells, and selective medium was added after the cells attached. Both Sf9 cells and High Five cells were incubated at 27°C. Protein expression was confirmed with SDS-PAGE and western blot. The protocol for building a stable cell line is according to the instruction manual.

### DNA Constructs

*Drosophila Dscam* (isoform 1-30-30-2) pcDNA3-V5-His [33] was fully resequenced and used as a template to generate different Dscam constructs: IgΔ1, IgΔ1–2, IgΔ1–5, and IgΔ1–7 with different Ig domains removed from the N-terminus and the intracellular domain removed. The full-length ectodomain construct is called FL-EC. The forward primers for different constructs were used with the same reverse primers in separate reactions to generate different N-terminus deletion Dscam constructs with a C-terminal extension containing a V5 epitope and the polyhistidine-tag encoded by pSecTag/FRT/V5-His vector (Fig 5A). For constructs with deletions of individual Ig domains (IgΔ3, IgΔ4, IgΔ1,4), EC-FL or IgΔ1 was used as the template, and the In-Fusion Cloning kit from Clontech (Mountain View, CA) was used to generate deletions according to the manufacturer’s recommended protocol. In brief, PCR primers were designed to flank the desired deletion region and contained 15-bp overlaps at their 5′ ends (Table 1); PCR products were gel-purified and recombined using the In-Fusion cloning reaction from the kit. The recombinant DNAs were transformed into *Escherichia coli* competent cells for screening. All of the constructs were sequenced using the ABI BigDye Terminator Cycle Sequencing Ready Reaction Kit v3.1. The reactions were run on an ABI3730 DNA Analyzer at the Nevada Genomics Center (UNR), and the sequences were analyzed using Vector NTI Advance 9 software (Life Technologies). To generate Myc-tagged full-length (Slit-FL) and the N-terminal cleavage fragment of Slit (Slit-N), pcDNA-Slit [26] was fully resequenced and subcloned into pSecTag/FRT-His TOPO vector. PCR was performed using different reverse primers with the same forward primer containing a Myc-tag nuclei acid sequence in the 5′ end (Table 1). To construct Myc-tagged EGF domains 1–3 of Slit, the same strategy was used (Table 1). pcDNA-slit-D was constructed by digesting pcDNA-slit with KpnI and EcoRV so that a 2,700 base pair fragment of the *slit* coding region was removed. This region was replaced with a synthetic gene fragment (Genscript, Piscataway, New Jersey) that lacked the LRR2 Robo binding site. pUAST-slit-D was constructed by sublconing a KpnI-XbaI fragment from pcDNA-slit-D. *Robo1ΔC* was constructed by PCR amplification of the Robo1 open reading frame from the initiating methionine to 9 amino acids after the end of the transmembrane domain (Table 1). EcoRI and XbaI sites in the primers allowed subcloning into pUAST.

### Cell Overlay Assays

COS-7 or 293 cells at 80% confluence were transfected with DNA expression constructs using Lipofectamine 2000 or 3000 (Life Technologies), according to the manufacturer’s instructions. For receptor expression, Dscam constructs were transfected into Cos-7 cells, and for ligand expression, pSegTag-Myc-Slit-FL and pSegTag-Myc-Slit-N were transfected into 293 cells. Approximately 48 h post-transfection, the supernatant was removed from receptor expressing cells and replaced with supernatant containing Myc-tagged ligand. Cells were incubated in a 37°C incubator for 1 to 2 h before being rinsed three times in 1XPBS and undergoing fixation and antibody labeling. Alternatively, the mixture of ligand and receptor DNA constructs (1:1) was cotransfected into COS-7 cells. Forty-eight hours post transfection, the medium was removed, and the cells were washed with PBS. After rinsing, the cells were fixed for 15 min in 4% paraformaldehyde with 0.1% Tween-20 at room temperature, followed by rinsing in PBS containing 0.1% Tween-20 (PBST) five times. The cells were blocked in 5% heat-denatured normal goat serum in PBST for 1 h. After blocking, the cells were incubated with primary antibodies diluted in 5% heat-denatured normal goat serum in PBST for 1 h: rabbit polyclonal anti-His tag at 1:1000, Abcam ab9018, or anti-Myc mouse monoclonal 1:500 from Abcam ab32. The cells were then rinsed five times in 1X PBST. Secondary antibodies, Alex 488 Goat anti-rabbit IgG, and/or Alex 595 Goat anti-mouse IgG (Jackson Labs) were diluted with 1:1000 in 5% heat-denatured goat serum in 1XPBST, then added to the cells, and incubated for 1 h at room temperature. The cells were subsequently washed in 1X PBST five times, followed by washing in 1X PBS and 1-min incubation in 4′,6-diamidino-2-phenylindole (DAPI) (Molecular Probes) to stain the nuclei. The cells were then washed in 1X PBS and mounted in FluorSave (Calbiochem). For double labeling, cells were incubated with the second primary and secondary antibody after finishing the first primary and secondary antibody with the same procedure.

### Immunoprecipitation Assays

Immunoprecipitation (IP) assays from cell culture followed the protocols of Banerjee et al. [67]. For IP assays with baculovirus proteins, the following protocol was followed: 50 μl of Protein G coupled beads were washed with high-salt binding buffer (100 mM phosphate buffer containing 1M NaCl, 10 U/ml Heparin, 1 mM DTT, 0.2 mM PMSF, and 1/1000 proteinase inhibitor cocktail) two times. The Sepharose beads were spun down at 500 x g for 2 min, and a magnet (Life Technologies) was used to separate the supernatant and Protein G Dynabeads. Two tubes of beads were used per IP: one for the ligand preclearing and the other for the IP itself. Eight soluble receptor Dscam-Fc supernatants from the High Five Dscam-Fc stable cell line and ligands (Slit-FL and Slit-N from the baculovirus expression) were collected by spinning down the culture at 1000 x g for 10 min, the supernatants were further concentrated using a centrifugal filter kit (Millipore), and finally, they were diluted in high-salt binding buffer. The ligand proteins were incubated with Protein G beads at room temperature for about 1 h to remove nonspecific binding. Two hundred and fifty μl of Dscam-Fc was incubated with 50 μl of prewashed Protein G Sepharose or Dynabeads rocking at RT for 1 h (because both proteins were expressed in the medium at 27°C). The unbound supernatant was removed, and the bead-receptor complex was incubated with precleared ligand for 1 h at room temperature. Nonbinding protein was removed, the beads were quickly washed in high-salt binding buffer three times, 50 μl sample buffer was added to each sample, and the samples were boiled for about 5 min and then either directly loaded into an SDS page gel or placed at −80°C. For IP assays from 293 and COS-7 cells, the cells were lysed 48 h after transfection in 500 μl prechilled cell lysis buffer (50 mM HEPES, pH 7.2, 100 mM NaCl, 1 mM MgCl2, 1 mM CaCl2, and 1% NP-40) containing Protease Inhibitors (10X, 100 μl/ml) and PMSF (0.3 M stock, 4 μl/ml). The lysates were kept on ice for 30 min and centrifuged at 15,000 g for 30 min at 4°C, followed by recentrifugation, and used subsequently for the IP. Protein G Plus-Agarose beads were used to pull down proteins; 50 μl beads for each IP reaction were washed three to five more times in 450 μl lysis buffer. The cell supernatants were precleared by incubation with Protein A beads. The beads were exchanged for anti-Robo antibody 13C9 (1:20 dilution) and incubated for 2 h at room temperature or 8 h at 4°C. The supernatant–antibody mix was incubated with prewashed Protein A or G beads for 1 h at the room temperature or 2 h at 4°C (Bhat et. al., 2010). The beads were then washed five times with lysis buffer and one time in cold PBS, followed by elution of the immunocomplexes in 50 μl SDS sample buffer, which was boiled at 100°C for 10 min, and then cooled in ice. Immunoblotting was with anti-V5 (1:1000 dilution, Life Technologies) to detect Dscam, and anti-SlitN antibody was used to detect Slit protein (1:200 dilution).

### Embryo Immunohistochemistry

The BP102 and 1D4 mAbs were obtained from the Developmental Studies Hybridoma Bank. *Drosophila* embryo stainings were performed as described in [68]. Anti-FasII staining was enhanced with Vectastain ABC (Vector Labs, Burlingame, California).

### Genetics

Standard genetic techniques were used to create recombinant and balanced stocks. The *NetAB; Dscam1* double mutant stock was created using the viable Netrin allele *NetAB*^*ΔGN*^, the hypomorphic *Dscam1*^*P*^ allele, *Dfd-GMR-nvYFP* marked *FM7*, and the *CyO wingless-lacZ* balancers.

### pCC Phenotype Analysis

For quantification of initial pCC axon behavior, early to mid-stage 13 embryos were used, as the growth rate defects of *slit-UC* make earlier quantification unreliable. Individual pCC axons were scored for midline crossing. For analysis of pCC crossing at the second commissure encountered, stage 14 embryos were used, and the presence of one or two axons from the pCC pathway at the CNS midline was scored as a positive crossing event for that segment. If an axon approached the midline and extended anteriorly along or near the midline, the axon was scored as crossing. In all cases, if it was unclear as to whether an axon was crossing, the axon was scored as not crossing. For quantification of longitudinal tracts, the number of tracts between segments was counted. The intersegment area was identified as lying between the intersegmental and segmental nerve roots and by a reduction of Fas2 staining in the cell bodies underneath the axon scaffold. Abdominal and thoracic segments were scored for all measures.

### Statistics

Statistical analysis was performed on Statistica (Dell Statistica). pCC midline projection errors and innervation of muscle 6/7 were recorded per individual embryo, and the data were analyzed using a Tukey HSD within a one-way ANOVA. Longitudinal connective and commissures stained with BP102 were scored as number thin or absent per embryo for a total of 11 segments scored per embryo.

## Supporting Information

S1 Data Quantification of fasciculation defects. The number of Fas2-positive fascicles present at the segment boundary in late stage 17 embryos was counted and expressed as an average per embryo (wild type equals six) for the indicated genotypes.
(XLSX)

S2 Data Quantification of pCC axon initial trajectories. The number of pCC axons crossing the midline in early to mid-stage 13 embryos was counted for each of the indicated genotypes.
(XLSX)

S3 Data Quantification of pCC axon trajectories after segment boundary crossing. The number of pCC axons crossing the midline after crossing the segment boundary for the first time was quantified in late stage 13 or early stage 14 embryos for the indicated genotypes.
(XLSX)

S4 Data Quantification of muscle 6/7 innervation in *slit* misexpression genotypes. Innervation of the muscle 6/7 cleft in late stage 17 embryos stained with anti-Fas2 was scored for the listed genotypes. Failure to innervate was recorded as a defect, and the total number of embryos examined is indicated (n).
(XLSX)

S5 Data Quantification of *slit* phenotype suppression by lateral *slit-N* expression. The presence of axons forming small circles (Fig 8) at the CNS midline was quantified (“circle”), and the total number of segments scored is also indicated.
(XLSX)

S6 Data Quantification of commissural axon defects in *Dscam1 NetAB* double mutants. Stage 16 or 17 embryos stained with BP102 were scored for reduced or absent commissures in the indicated genotypes. The abdominal and thoracic segments were scored (11 per embryo).
(XLSX)

S7 Data Quantification of longitudinal defects in *Dscam1 NetAB* double mutants. Stage 16 or 17 embryos stained with BP102 were scored for reduced or absent longitudinal connectives in the indicated genotypes. The abdominal and thoracic segments were scored (11 per embryo).
(XLSX)

S1 Fig Late-stage development series of *robo1* and *Dscam1 robo1* nerve cords. Staged nerve cords stained with BP102 to reveal the axon scaffold. (A) *robo*^*4*^ allele from mid-stage 16 to late stage 17 as labeled (e = early, l = late). The *robo* phenotype maintains a regular appearance as the embryo ages. (B) *Dscam*^*P*^ *robo*^*4*^ double mutant with the same stages as in A. The phenotype is quite variable from segment to segment within each nerve cord, and this increases with age. The number of segments visible per panel is less, suggesting that condensation of the nerve cord has not occurred. The nerve cord is also thinner and distorted. Note how the longitudinal connectives are frequently missing or collapsed into one connective.
(TIF)

S2 Fig Late stage 17 embryos stained with anti-Fas2 (1D4 monoclonal) to reveal the longitudinal tracts. (A) Wild-type embryo displaying the Fas2 longitudinal tracts running parallel to the CNS midline. (B) *robo2*^*lea-2*^ mutant with disruptions to the outermost fascicles (arrows) and occasional defects in the innermost fascicle (arrowhead). (C) *Dscam1*^*1*^ *robo2*^*lea-2*^ double mutant showing a strong overall disruption to all fascicles, including the outermost (arrows) and innermost (arrowheads). (D) *Dscam1*^*1*^ *Dscam3*^*c02862*^ double mutant in which the longitudinals are disorganized, inappropriately approaching the midline or forming *robo*-like circles (arrowheads) and displaying breaks in the outermost fascicle (arrows).
(TIF)

S3 Fig Schematic of pCC axon behavior in different genotypes. Cartoons representing the behavior of the pCC ascending and MP1 descending longitudinal pioneer axons in wild type, *slit-UC*, and *robo* mutants. Commissural axons are shown as light gray horizontal bars. The pCC axon is brown, MP1 scarlet. Additional neurons include the SP1 commissural pioneers (light blue), the aCC motor neuron (light gray), dMP2 (yellow), and vMP2 (medium gray). (A) In wild-type embryos, both pathways grow away from the midline and fasciculate upon meeting. Both pathways subsequently defasciculate to avoid growth towards the midline. (B) In the *slit-UC* mutant, the axon trajectories are largely normal, although 30% of pCC axons cross the midline shortly after starting growing. The majority of axons do not cross the midline at the first commissure. Almost all of the ascending and descending pioneers cross the midline at the next commissure encountered, and pCC axons frequently grow over the pCC cell body. The MP1 axons are frequently found at the anterior edge of the commissure. This combined behavior creates the circles seen in the *robo* mutant. The critical difference is that only a minority of pCC axons cross early in their trajectories. The cell bodies are shown in their wild-type positions for clarity, but in *slit-UC* mutants, they are usually closer to the midline in a manner resembling *robo* mutants. (C) In *robo* mutants, almost all pCC axons cross the midline (red arrow) at the first commissure they encounter. They almost always recross the midline in the next commissure they encounter. The descending MP1 axons also incorrectly cross the midline after crossing the segment boundary, creating characteristic axon circles.
(TIF)

S4 Fig Expression and purification of baculovirus Slit. In all experiments, High Five cells were transfected with Baculovirus Slit-FL or Slit-N and cultured at 27°C. Culture medium was centrifuged, and the supernatant concentrated in a centrifugal filter unit. Equal volumes from each condition were loaded into an SDS-PAGE gel. Immunoblot analysis was with anti-His antibody, which detects the 6xHis epitope tag at the carboxy-terminal of Slit. (A) Full-length Slit was expressed at various multiplicities of infection (MOI = ratio of infectious virus particles to cells) using the Bac to Bac HBM baculovirus expression system. Immunoblot analysis found MOIs of 2–5 to be most effective. (B) Full-length Slit was expressed at MOI = 2 and harvested at different post-infection (PI) times: 24, 48, 72, and 96 h. Those found to be the most effective were 48 and 72 h PI times. (C) N-terminal Slit was expressed at different MOIs, with MOI = 2 being the most productive. (D) Slit-FL was further purified over a nickel column to bind the 6xHis epitope tag and analyzed by Coomassie Blue staining. The full-length Slit fragment (~178 kD) is clearly visible. (E) Slit-FL purification analyzed on an immunoblot with anti-Slit-N antibody. There is a lesser amount of Slit-N, which is presumably purified by dimerization with Slit-FL, as it lacks the carboxy-terminal epitope tag.
(TIF)

S5 Fig Embryonic expression of Dscam1 protein. Stage 16 embryo stained with an antibody against Dscam1. The DAB staining was enhanced with nickel to increase weak staining. The CNS axon scaffold is at the bottom of the picture. There appear to be some unstained fascicles in the commissures, but otherwise, we estimate that 95% of CNS axons are stained. The nerve roots containing the motor neurons in the process of navigating towards their target muscles are also Dscam1 positive, and staining can be seen in the focal plane underneath the muscles (arrows). Innervation of muscles 6, 7, 12, and 13 will not occur until the end of the next stage. The muscle attachment sites also stain strongly (arrowheads). We have tested antibodies from both the Zipursky and Schmucker laboratories and only see this staining with one antibody. In *Dscam1*^*1*^ null alleles, axonal staining completely disappears, but the muscle attachment site staining is reduced but not absent. We therefore cannot exclude the possibility that the muscles or the tendon cells express a low level of Dscam1.
(TIF)

S6 Fig Disruption of the CNS in *NetAB; Dscam1* double mutants. Stage 16–17 *NetAB; Dscam1* embryos stained with BP102 to reveal the CNS axon scaffold and the range of defects seen in this double mutant.
(TIF)

## Abbreviations

CNS: central nervous system
DAPI: 4′,6-diamidino-2-phenylindole
EC: ectodomain
EGF: epidermal growth factor
FL: full-length
HSD: honest significant difference
Ig: immunoglobulin
IP: immunoprecipitation
ISNb: intersegmental nerve b
LRR: leucine-rich repeat
mAb: monoclonal antibody
MOI: multiplicity of infection
NIH: National Institutes of Health
PBST: phosphate buffered saline with Tween
PI: post infection
PMSF: phenylmethylsulfonyl fluoride
