# Supplementary figures and images for: Dscam1 Forms a Complex with Robo1 and the N-Terminal Fragment of Slit to Promote the Growth of Longitudinal Axons
Source: PLoS Biol. 2016 Sep 21;14(9):e1002560. doi: 10.1371/journal.pbio.1002560 (PMC5031454; doi:10.1371/journal.pbio.1002560)

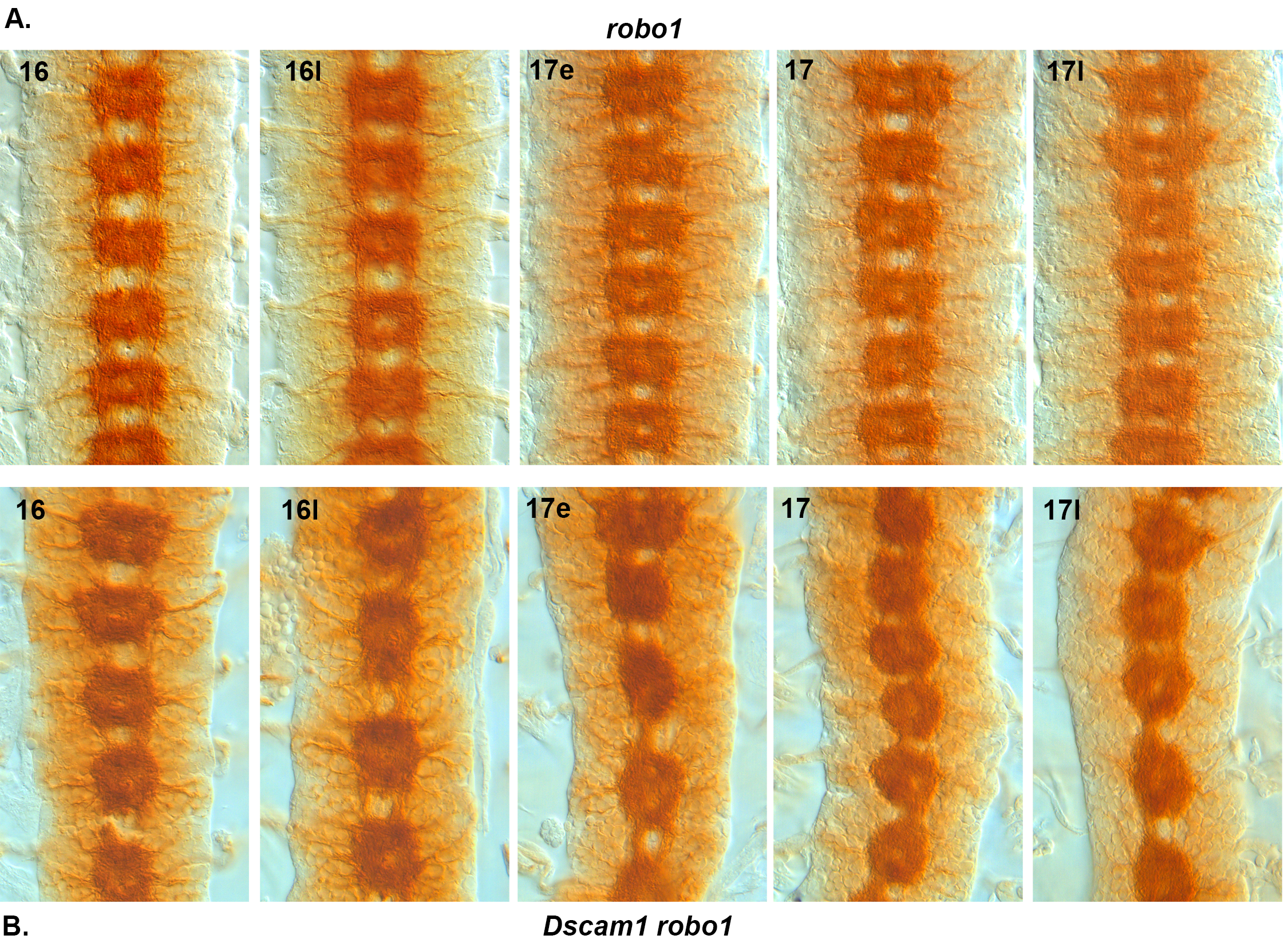

Supplement: S1 Fig — Staged nerve cords stained with BP102 to reveal the axon scaffold. (A) robo4 allele from mid-stage 16 to late stage 17 as labeled (e = early, l = late). The robo phenotype maintains a regular appearance as the embryo ages. (B) DscamP robo4 double mutant with the same stages as in A. The phenotype is quite variable from segment to segment within each nerve cord, and this increases with age. The number of segments visible per panel is less, suggesting that condensation of the nerve cord has not occurred. The nerve cord is also thinner and distorted. Note how the longitudinal connectives are frequently missing or collapsed into one connective. (TIF) [file pbio.1002560.s008.tif]

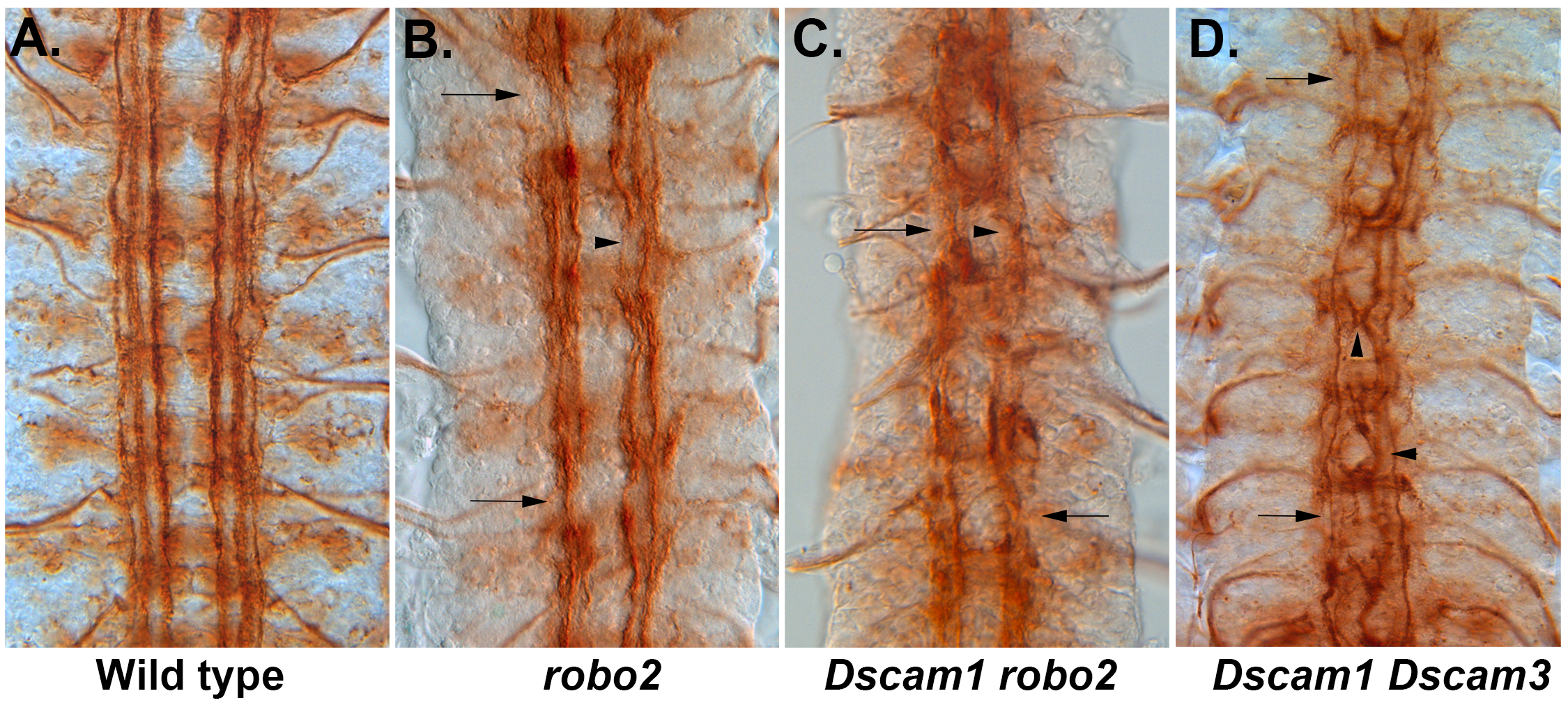

Supplement: S2 Fig — (A) Wild-type embryo displaying the Fas2 longitudinal tracts running parallel to the CNS midline. (B) robo2lea-2 mutant with disruptions to the outermost fascicles (arrows) and occasional defects in the innermost fascicle (arrowhead). (C) Dscam11 robo2lea-2 double mutant showing a strong overall disruption to all fascicles, including the outermost (arrows) and innermost (arrowheads). (D) Dscam11 Dscam3c02862 double mutant in which the longitudinals are disorganized, inappropriately approaching the midline or forming robo-like circles (arrowheads) and displaying breaks in the outermost fascicle (arrows). (TIF) [file pbio.1002560.s009.tif]

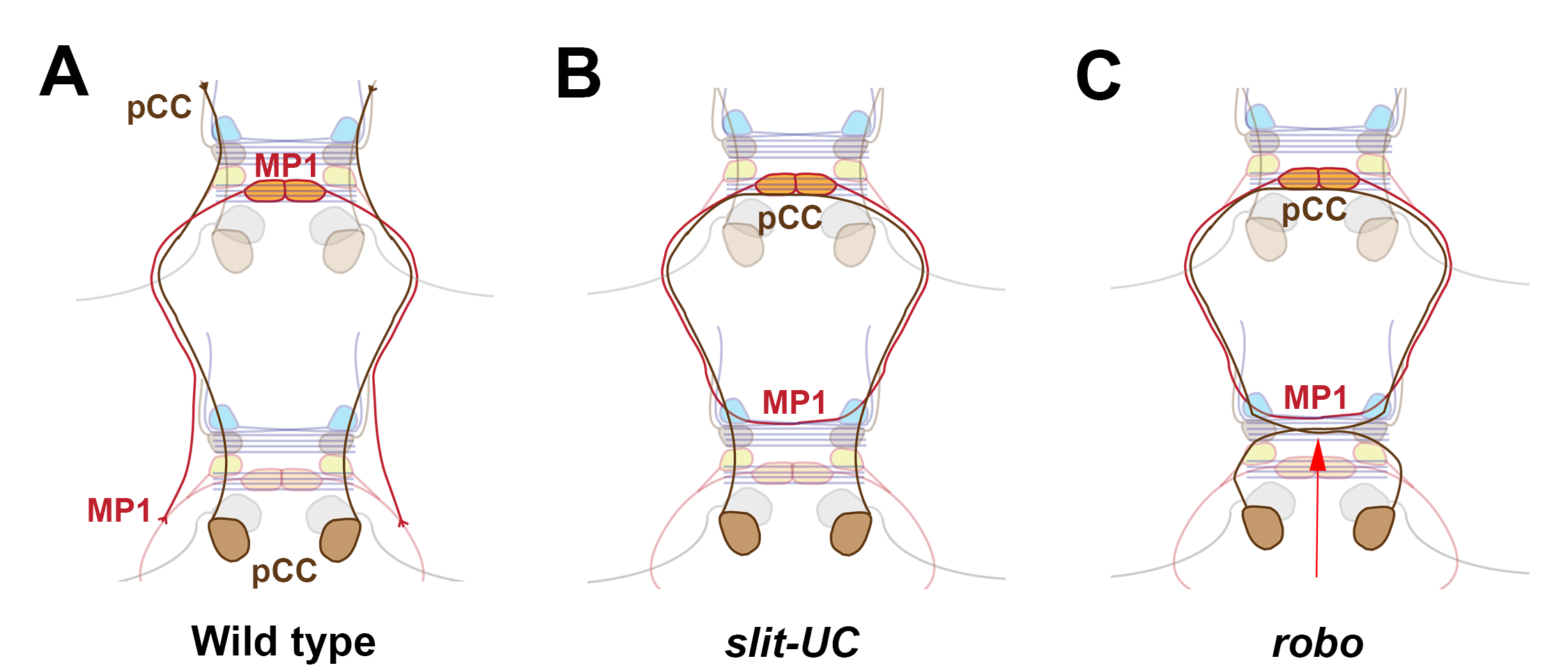

Supplement: S3 Fig — Cartoons representing the behavior of the pCC ascending and MP1 descending longitudinal pioneer axons in wild type, slit-UC, and robo mutants. Commissural axons are shown as light gray horizontal bars. The pCC axon is brown, MP1 scarlet. Additional neurons include the SP1 commissural pioneers (light blue), the aCC motor neuron (light gray), dMP2 (yellow), and vMP2 (medium gray). (A) In wild-type embryos, both pathways grow away from the midline and fasciculate upon meeting. Both pathways subsequently defasciculate to avoid growth towards the midline. (B) In the slit-UC mutant, the axon trajectories are largely normal, although 30% of pCC axons cross the midline shortly after starting growing. The majority of axons do not cross the midline at the first commissure. Almost all of the ascending and descending pioneers cross the midline at the next commissure encountered, and pCC axons frequently grow over the pCC cell body. The MP1 axons are frequently found at the anterior edge of the commissure. This combined behavior creates the circles seen in the robo mutant. The critical difference is that only a minority of pCC axons cross early in their trajectories. The cell bodies are shown in their wild-type positions for clarity, but in slit-UC mutants, they are usually closer to the midline in a manner resembling robo mutants. (C) In robo mutants, almost all pCC axons cross the midline (red arrow) at the first commissure they encounter. They almost always recross the midline in the next commissure they encounter. The descending MP1 axons also incorrectly cross the midline after crossing the segment boundary, creating characteristic axon circles. (TIF) [file pbio.1002560.s010.tif]

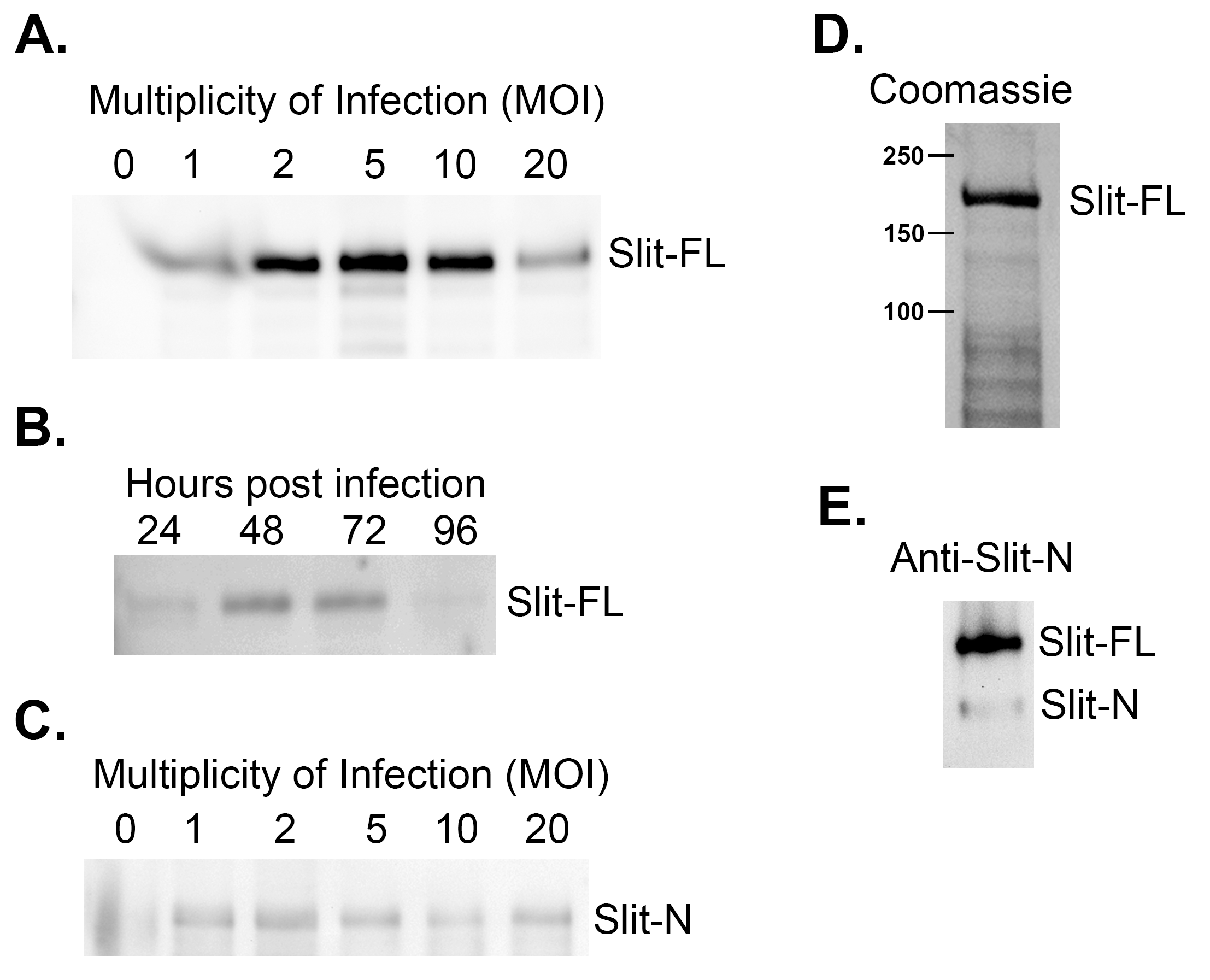

Supplement: S4 Fig — In all experiments, High Five cells were transfected with Baculovirus Slit-FL or Slit-N and cultured at 27°C. Culture medium was centrifuged, and the supernatant concentrated in a centrifugal filter unit. Equal volumes from each condition were loaded into an SDS-PAGE gel. Immunoblot analysis was with anti-His antibody, which detects the 6xHis epitope tag at the carboxy-terminal of Slit. (A) Full-length Slit was expressed at various multiplicities of infection (MOI = ratio of infectious virus particles to cells) using the Bac to Bac HBM baculovirus expression system. Immunoblot analysis found MOIs of 2–5 to be most effective. (B) Full-length Slit was expressed at MOI = 2 and harvested at different post-infection (PI) times: 24, 48, 72, and 96 h. Those found to be the most effective were 48 and 72 h PI times. (C) N-terminal Slit was expressed at different MOIs, with MOI = 2 being the most productive. (D) Slit-FL was further purified over a nickel column to bind the 6xHis epitope tag and analyzed by Coomassie Blue staining. The full-length Slit fragment (~178 kD) is clearly visible. (E) Slit-FL purification analyzed on an immunoblot with anti-Slit-N antibody. There is a lesser amount of Slit-N, which is presumably purified by dimerization with Slit-FL, as it lacks the carboxy-terminal epitope tag. (TIF) [file pbio.1002560.s011.tif]

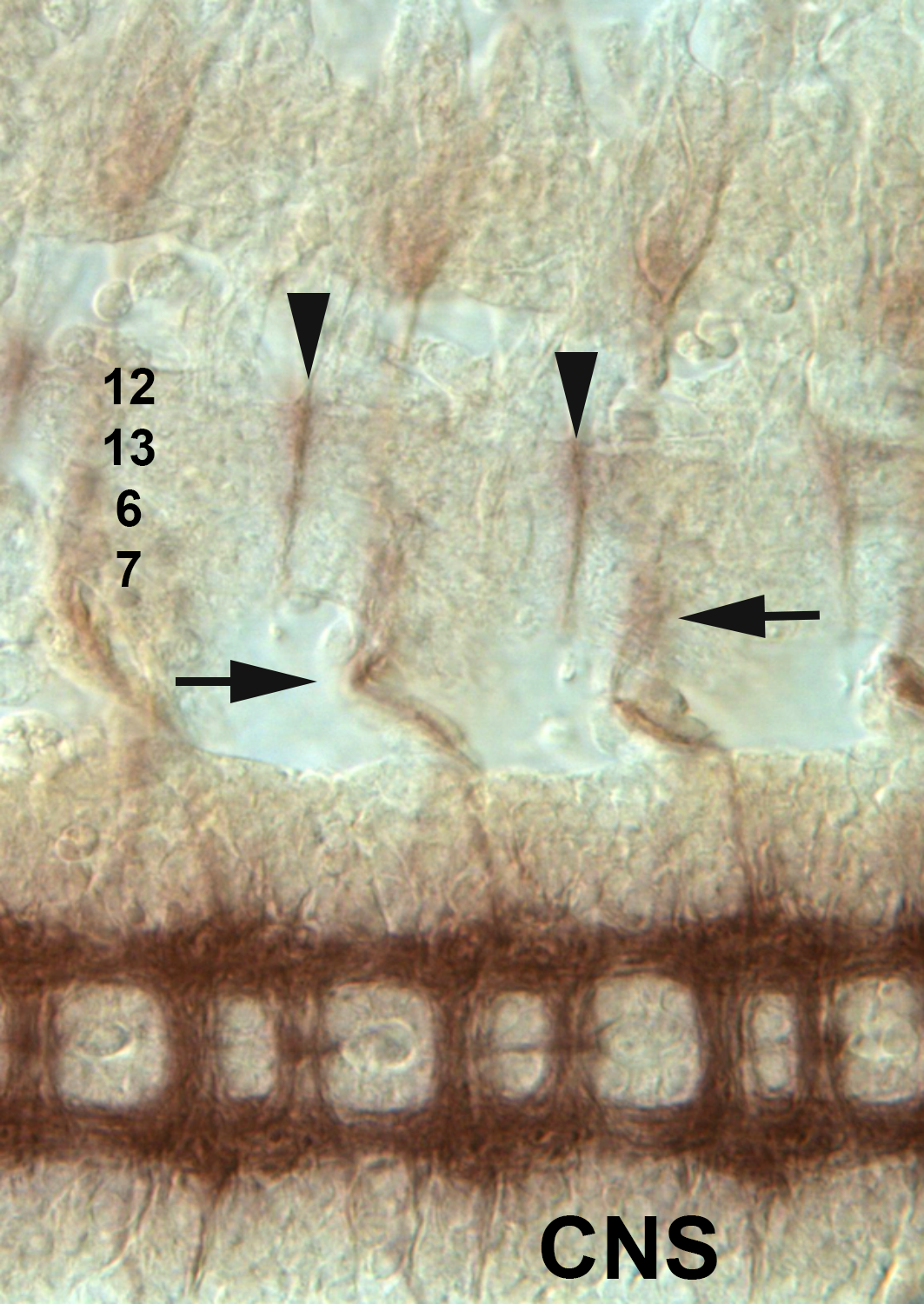

Supplement: S5 Fig — Stage 16 embryo stained with an antibody against Dscam1. The DAB staining was enhanced with nickel to increase weak staining. The CNS axon scaffold is at the bottom of the picture. There appear to be some unstained fascicles in the commissures, but otherwise, we estimate that 95% of CNS axons are stained. The nerve roots containing the motor neurons in the process of navigating towards their target muscles are also Dscam1 positive, and staining can be seen in the focal plane underneath the muscles (arrows). Innervation of muscles 6, 7, 12, and 13 will not occur until the end of the next stage. The muscle attachment sites also stain strongly (arrowheads). We have tested antibodies from both the Zipursky and Schmucker laboratories and only see this staining with one antibody. In Dscam11 null alleles, axonal staining completely disappears, but the muscle attachment site staining is reduced but not absent. We therefore cannot exclude the possibility that the muscles or the tendon cells express a low level of Dscam1. (TIF) [file pbio.1002560.s012.tif]

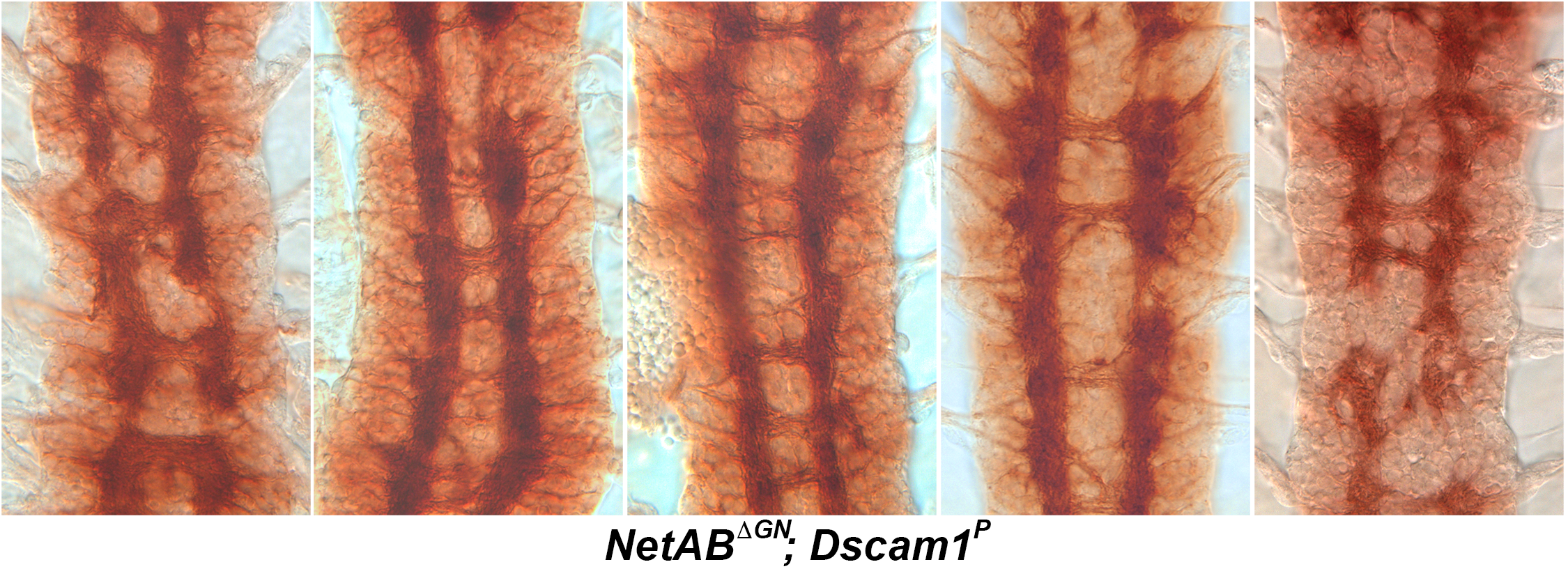

Supplement: S6 Fig — Stage 16–17 NetAB; Dscam1 embryos stained with BP102 to reveal the CNS axon scaffold and the range of defects seen in this double mutant. (TIF) [file pbio.1002560.s013.tif]
